# Extending the Functional Subnetwork Approach to a Generalized Linear Integrate-and-Fire Neuron Model

**DOI:** 10.3389/fnbot.2020.577804

**Published:** 2020-11-13

**Authors:** Nicholas S. Szczecinski, Roger D. Quinn, Alexander J. Hunt

**Affiliations:** ^1^Department of Mechanical and Aerospace Engineering, West Virginia University, Morgantown, WV, United States; ^2^Department of Mechanical and Aerospace Engineering, Case Western Reserve University, Cleveland, OH, United States; ^3^Department of Mechanical and Materials Engineering, Portland State University, Portland, OR, United States

**Keywords:** spiking neuron, generalized integrate and fire models, non-spiking neuron, neurorobotics, functional subnetwork approach, synthetic nervous system

## Abstract

Engineering neural networks to perform specific tasks often represents a monumental challenge in determining network architecture and parameter values. In this work, we extend our previously-developed method for tuning networks of non-spiking neurons, the “Functional subnetwork approach” (FSA), to the tuning of networks composed of spiking neurons. This extension enables the direct assembly and tuning of networks of spiking neurons and synapses based on the network's intended function, without the use of global optimization or machine learning. To extend the FSA, we show that the dynamics of a generalized linear integrate and fire (GLIF) neuron model have fundamental similarities to those of a non-spiking leaky integrator neuron model. We derive analytical expressions that show functional parallels between: (1) A spiking neuron's steady-state spiking frequency and a non-spiking neuron's steady-state voltage in response to an applied current; (2) a spiking neuron's transient spiking frequency and a non-spiking neuron's transient voltage in response to an applied current; and (3) a spiking synapse's average conductance during steady spiking and a non-spiking synapse's conductance. The models become more similar as additional spiking neurons are added to each population “node” in the network. We apply the FSA to model a neuromuscular reflex pathway two different ways: Via non-spiking components and then via spiking components. These results provide a concrete example of how a single non-spiking neuron may model the average spiking frequency of a population of spiking neurons. The resulting model also demonstrates that by using the FSA, models can be constructed that incorporate both spiking and non-spiking units. This work facilitates the construction of large networks of spiking neurons and synapses that perform specific functions, for example, those implemented with neuromorphic computing hardware, by providing an analytical method for directly tuning their parameters without time-consuming optimization or learning.

## 1. Introduction

Neuromorphic computing hardware is becoming more widely available (Khan et al., 2008; Pfeil et al., 2013; Benjamin et al., 2014; Gehlhaar, 2014; Merolla et al., 2014; Ionica and Gregg, 2015; Davies et al., 2018). Such chips have non-traditional architecture, with highly-parallel processing and specialized circuits that mimic neural and synaptic dynamics. These chips mimic the communication of spiking neural networks, whose discrete communication events (i.e., spikes) reduce the communication overhead relative to continuous networks. Many canonical brain networks have been tested with these chips including decorrelation networks, winner-take-all networks, and balanced random networks (Pfeil et al., 2013), as well as other networks that perform complex computations, such as multi-object recognition (Merolla et al., 2014) and keyword-matching (Blouw et al., 2018), using <100 mW of power in the process.

Neuromorphic hardware is advancing both computational neuroscience (Eliasmith and Anderson, 2002; Eliasmith et al., 2012) and artificial intelligence (Pfeil et al., 2013; Benjamin et al., 2014; Merolla et al., 2014), and soon will play a critical role in robotics. Animals' mobility shows that neuron-based control is effective, and several groups have already developed neural-inspired controllers that could benefit from the low power and parallel computing of neuromorphic hardware (Ayers et al., 2010; Floreano et al., 2014; Dasgupta et al., 2015; Hunt et al., 2017; Szczecinski and Quinn, 2017b; Dürr et al., 2019). However, to apply these neuromorphic chips to robotics, these controllers must be converted into a chip's specific neural model, which may not be trivial. All chips use a variant of the integrate-and-fire model (Brunel and van Rossum, 2007). Toward this goal, we have developed methods for applying our functional subnetwork approach (FSA) for designing non-spiking recurrent neural networks (Szczecinski et al., 2017b) to the specific generalized integrate-and-fire (GLIF) model used by Intel's Loihi chip (Mihalaş and Niebur, 2009; Davies et al., 2018). We will show that these models (i.e., non-spiking and GLIF) have several parallels that enable a network designer to map between them. The details of this comparison are listed at the end of the Introduction.

Setting parameter values in dynamic neural networks can be extremely difficult. Even when network architecture is created directly from animal architecture, parameters cannot be practically measured across all neurons and synapses, and there may be thousands of parameters that dynamically interact. Therefore, these parameter values must be set by the modeler such that the network produces the desired behavior. Some methods and tools have been developed to assist neural designers when mapping network behavior to a desired output, for example, the Neuroengineering Framework and its browser-based design program, Nengo (Eliasmith and Anderson, 2002; Maass and Markram, 2004; Bekolay et al., 2014). These methods seek to build populations of neurons, whose average activity (i.e., spiking frequency) encodes a value of interest. Each population can then interact with others to perform specific operations, such as arithmetic or calculus. This technique is very powerful, enabling the construction of brain-scale networks (Eliasmith, 2013). However, one drawback is that within each population, the connectivity is random, and may not provide insight into how biological networks are structured at small scales. This method is also not ideal for modeling networks with relatively few neurons, such as those that have been described in the locomotion networks of animals (e.g., Bueschges et al., 1994; Sauer et al., 1996; Berg et al., 2015).

As an alternative to this technique we have developed methods for explicitly computing neural and synaptic parameter values for non-spiking dynamical neural networks that perform arithmetic and calculus (Szczecinski et al., 2017b). Such networks are also called “recurrent neural networks,” because each neuron's instantaneous state is a function of its own history, producing a form of self-feedback. While such recurrent dynamics can make it difficult to tune networks, such continuous dynamical neural models enable direct analysis of a network's eigenvalues, equilibrium points, and therefore, individual neuron behavior in response to specific inputs (Szczecinski et al., 2017a,b). Such analysis can be difficult to perform on spiking networks, but is particularly important in robotics, in which engineers seek to guarantee a robot's stability and the controller's robustness to parameter changes or sensor noise. The resulting networks are sparse and based on known anatomy, similar to related robotic controllers composed of analog very large scale integration (VLSI) circuits or efficient, discrete-time neuron models (Ayers and Crisman, 1993; Ayers et al., 2010).

Such non-spiking, continuous-valued models theoretically have the same activation dynamics as the average spiking frequency of a population of spiking neurons, all of whom receive the same (noisy) inputs (Wilson and Cowan, 1972). The current manuscript explores this assertion by identifying relationships between the parameter values in the non-spiking model used in our previous work (Szczecinski et al., 2017a,b) and those in a GLIF model (Mihalaş and Niebur, 2009; Davies et al., 2018). Applying the functional subnetwork approach to spiking networks has three primary benefits: First, it enables rapid and direct assembly of spiking networks that have predictable performance; second, it enhances neural robot controllers with richer dynamics than recurrent neural networks; and third, it is a tool for implementing neural controllers on neuromorphic hardware for robots.

In this manuscript, we demonstrate the first of these benefits by extending our previously-developed design tools for non-spiking models (Szczecinski et al., 2017b) to spiking models. We present three “parallels” between the non-spiking and spiking models that enable the extension of our non-spiking network design techniques to spiking networks. This extension includes reducing the impact of non-linear relationships within a network. We derive three parallels between these models:

P1. The steady-state spiking frequency of a spiking neuron is parallel to the steady-state depolarization of a non-spiking neuron because each is proportional to the current applied to the neuron. We refer to both of these quantities as the “activation” of the neuron. The activation of each model can be related to the other via model parameters, and specific parameter values increase the similarity between spiking frequency and non-spiking depolarization.
P2. The instantaneous spiking frequency of a spiking neuron is parallel to the instantaneous depolarization of a non-spiking neuron. Each exhibits a transient response when stimulated. The decay rate of each model can be related to the other via model parameters, and specific parameter values increase the similarity between the spiking frequency time constant and the non-spiking membrane time constant.
P3. The time-averaged conductance of a spiking synapse is parallel to the conductance of a non-spiking synapse because each is proportional to the activation of the pre-synaptic neuron. Both spiking and non-spiking synapses can be designed to implement a given “gain” value, i.e., the ratio between the post-synaptic (i.e., receiving) and pre-synaptic (i.e., sending) neurons' activations. Specific parameter values increase the similarity between the time-averaged spiking synapse conductance and the non-spiking synapse conductance.

This manuscript is organized as follows. The methods in section 2 present the non-spiking and GLIF models and compute fundamental quantities for each, including equilibrium points and useful relationships between parameter values and variables. We use these expressions to extend our FSA for designing non-spiking networks to spiking models. The results in section 3 demonstrate parallels P1-P3 and leverage them into a sequential process for designing a spiking pathway. In section 4, the results from section 3 are applied to a neuromuscular model of a stretch reflex, and the resulting motion of the models is compared. Finally, the discussion in section 5 summarizes the work, explains how neurobiologists and roboticists can apply this work to their research, and proposes future work. To aid the reader, variable names are defined in Table 1.

**Table 1:** List of variables and descriptions.

**Variable**	**Description**
**NON-SPIKING**	
$\bar{U}$	Membrane voltage, state variable
${\overset{-}{C}}_{mem}$	Membrane capacitance, constant parameter
*G*_*mem*_	Membrane conductance/leak conductance, constant parameter
*I*_*app*_	Applied current, input variable
$\bar{G}$_*max*_	Maximum non-spiking synaptic conductance, constant parameter
$\overset{-}{G_{s}}$	Instantaneous non-spiking synaptic conductance, piecewise linear function of the pre-synaptic neuron's voltage
$\overset{-}{E_{s}}$	Non-spiking synaptic reversal potential, constant parameter
**SPIKING**	
*U*	Membrane voltage, state variable
θ	Spiking threshold, state variable
*C*_*mem*_	Membrane capacitance, constant parameter
*G*_*mem*_	Membrane conductance/leak conductance, constant parameter
*I*_*app*_	Applied current, input variable
θ_0_	Initial spiking threshold, constant parameter
τ_θ_	Spiking threshold time constant, constant parameter
*m*	Proportionality constant that determines the change in θ relative to *U*, constant parameter
*G*_*max*_	Maximum spiking synaptic conductance, constant parameter
*G*_*s*_	Instantaneous synaptic conductance, state variable
τ_*s*_	Synaptic time constant, constant parameter
*E*_*s*_	Spiking synaptic reversal potential, constant parameter

## 2. Methods

In this section, we present both the non-spiking model and the spiking model. For each, we compute parameter values necessary to demonstrate parallels P1–P3. Then we briefly summarize the philosophy behind the FSA.

### 2.1. Non-spiking Neuron and Synapse Models

**Figure 1:**
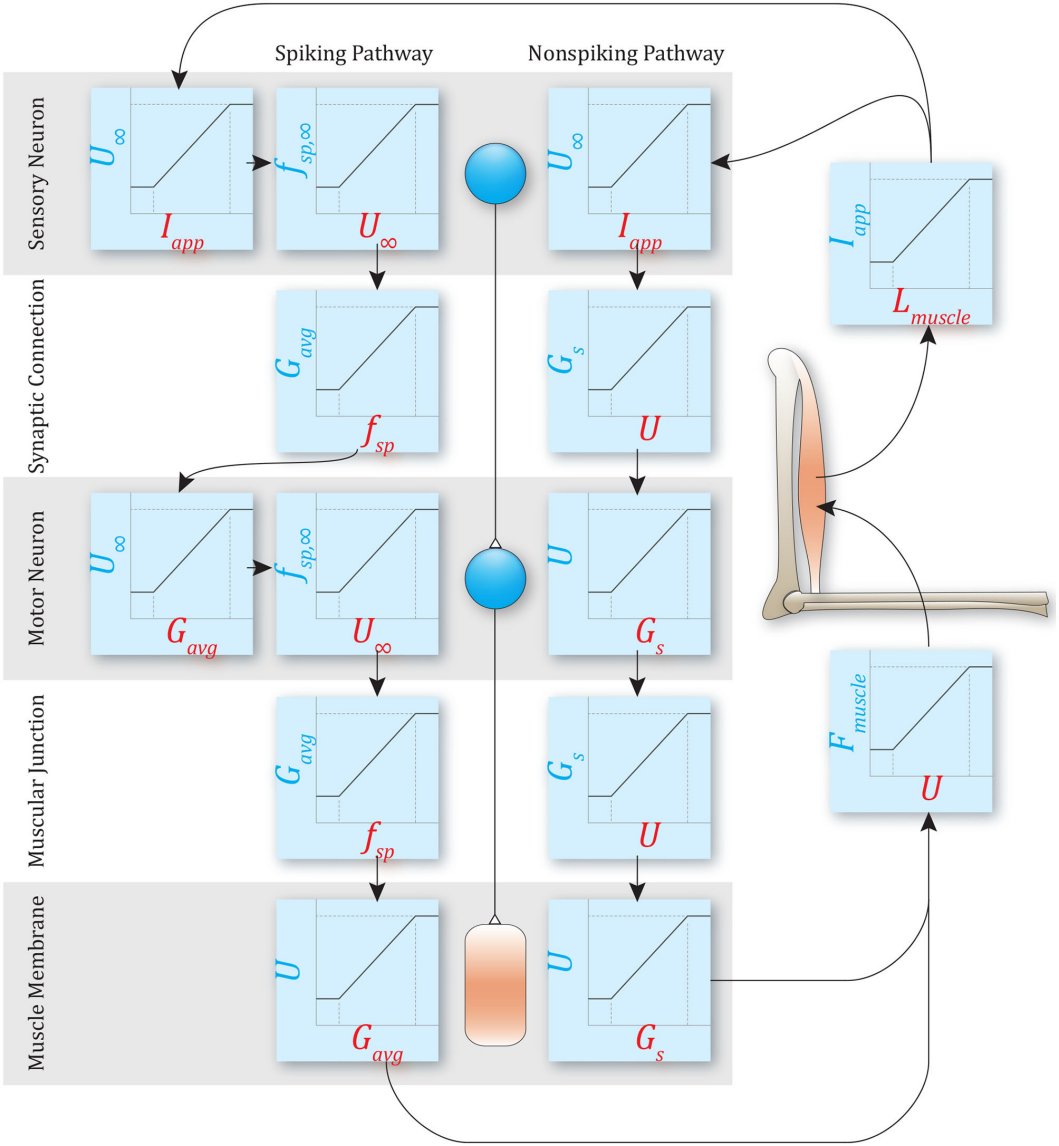
An example of a simple reflex pathway that encodes muscle stretch and decodes to muscle force. Both non-spiking and spiking implementations are shown. Both methods require mapping to and from mechanical states and neural states, but the specific transforms required depend on the model used. The rest of this manuscript demonstrates the relationships between parameters in these models.

The non-spiking model is a leaky integrator, or recurrent neural model (Beer and Gallagher, 1992). Such a model describes the subthreshold dynamics of a neuron with the differential equation

(1) $$\begin{array}{l} {\overset{-}{C}}_{mem}\cdot \frac{d\bar{U}}{dt}=-G_{mem}\cdot \bar{U}+\overset{n}{\underset{i=1}{\sum }}{\bar{G}}_{s,i}\cdot \left(E_{s,i}-\bar{U}\right)+I_{app}+I_{bias}, \end{array}$$

where $\bar{U}$ is the non-spiking neuron voltage above its rest potential (referred to as “membrane depolarization” throughout, see Szczecinski et al., 2017b for more detail), ${\overset{-}{C}}_{mem}$ is the capacitance of the cell membrane, *G*_*mem*_ is the leak conductance, $\bar{G}$_*s,i*_ is the instantaneous conductance of the *i*th incoming non-spiking (i.e., graded) synapse, *E*_*s,i*_ is the reversal potential of the *i*^*th*^ incoming synapse relative to the neuron's rest potential, *I*_*app*_ is the applied current that encodes information (e.g., muscle stretch, as shown in Figure 1), and *I*_*bias*_ is the constant offset current. The synaptic conductance $\bar{G}$_*s*_ is a piecewise linear function of the pre-synaptic neuron voltage,

(2) $$\begin{array}{l} {\bar{G}}_{s}={\bar{G}}_{max,i}\cdot \{\begin{array}{cc} 0, & \text{ if }{\bar{U}}_{pre}\leq 0,\\ \frac{{\bar{U}}_{pre}}{R}, & \text{ if }0<{\bar{U}}_{pre}<R,\\ 1, & \text{ if }{\bar{U}}_{pre}\geq R. \end{array} \end{array}$$

$\bar{G}$_*max*_ is the maximum synaptic conductance, and *R* is the maximum membrane depolarization of neurons in the network (Szczecinski et al., 2017b). Parameters with a bar (e.g., $\bar{U}$) are those that relate to the non-spiking model, and will be mapped to analogous parameters in the spiking model in section 2.2. The results in section 3 explain how to map from these values into their spiking model counterparts.

The steady-state membrane depolarization, $\bar{U}$_∞_, is calculated by solving Equation (1) when *d*$\bar{U}$/*dt* = 0,

(3) $$\begin{array}{l} {\bar{U}}_{\infty }=\frac{\sum_{i=1}^{n}{\bar{G}}_{s,i}\cdot E_{s,i}+I_{app}+I_{bias}}{\sum_{i=1}^{n}{\bar{G}}_{s,i}+G_{mem}}. \end{array}$$

Note that the equilibrium voltage is effectively the average of incoming synaptic potentials *E*_*s*_, weighted by their conductances $\bar{G}$_*s*_. If a neuron receives an applied current but has no incoming synaptic connections, this expression simplifies to:

(4) $$\begin{array}{l} {\bar{U}}_{\infty }=\frac{I_{app}+I_{bias}}{G_{mem}}. \end{array}$$

If *I*_*bias*_ = 0, then $\bar{U}$ is directly proportional to *I*_*app*_. This expression will be used to demonstrate parallel P1 between the models.

How does the non-spiking model's response evolve over time? The response to a tonic current that is turned on at time *t* = 0 is

(5) $$\begin{array}{l} \bar{U}\left(t\right)={\bar{U}}_{\infty }\cdot \left(1-e^{-t/\tau_{mem}}\right), \end{array}$$

where *U*_∞_ comes from Equation (3), and

(6) $$\begin{array}{l} \tau_{mem}=\frac{C_{mem}}{\sum_{i=1}^{n}G_{s,i}+G_{mem}}. \end{array}$$

The transient response of $\bar{U}$ decays with time constant τ_*mem*_, which will be compared to the spiking model's transient spiking frequency, demonstrating parallel P2 between the models.

The effect of a synaptic input on the post-synaptic neuron can be quantified by the “gain” of the synapse. We define the gain *k*_*syn*_ = $\bar{U}$_∞,*post*_/$\bar{U}$_∞,*pre*_, the ratio between the post-synaptic and pre-synaptic steady-state voltage. Substituting the expression for synaptic conductance in Equation (2) into Equation (3) relates the synapse's parameter values to its gain (Szczecinski et al., 2017b):

(7) $$\begin{array}{l} {\bar{G}}_{max}=\frac{k_{syn}\cdot R}{E_{s}-k_{syn}\cdot R}. \end{array}$$

This expression will be extended to design spiking synapses, once we demonstrate parallel P3 between the models.

### 2.2. Spiking Neuron and Synapse Model

The spiking model used in this work is a generalized leaky integrate and fire (GLIF) model (Mihalaş and Niebur, 2009; Davies et al., 2018). The dynamics of the membrane voltage are identical to Equation (1) (Equation 8), except that *U* is reset to 0 after crossing the spiking threshold, θ (Equation 10). In addition, the threshold θ is itself a dynamical variable that evolves according to Equation (9). The dynamics of *U* and θ interact to produce a diverse set of spiking responses (Mihalaş and Niebur, 2009).

The spiking model's dynamics are:

(8) $$\begin{array}{l} C_{mem}\cdot \frac{dU}{dt}=-G_{mem}\cdot U+\overset{n}{\underset{i=1}{\sum }}G_{s,i}\cdot \left(E_{s,i}-U\right)+I_{app}+I_{bias} \end{array}$$

(9) $$\begin{array}{l} \tau_{\theta }\cdot \frac{d\theta }{dt}=-\theta +\theta_{0}+m\cdot U \end{array}$$

(10) $$\begin{array}{ll} \text{if }U & \geq \theta ,0\leftarrow U, \end{array}$$

where the variable names and functions are the same as in Equation (1), with the exception of the synaptic conductance *G*_*s,i*_ (described below); and the threshold variable θ, whose equation includes the threshold time constant τ_θ_, the initial threshold θ_0_, and the proportionality constant that specifies how θ changes in response to changes in *U*, called *m*.

The spiking synapse conductance *G*_*s*_ is reset to its maximum value *G*_*max*_ when the pre-synaptic neuron spikes, and decays to 0 with a time constant τ_*s*_,

(11) $$\begin{array}{l} \tau_{s}\cdot \frac{dG_{s}}{dt}=-G_{s}. \end{array}$$

The steady-state threshold value θ_∞_ is calculated by solving Equation (9) when *dθ*/*dt* = 0,

(12) $$\begin{array}{l} \theta_{\infty }=\theta_{0}+m\cdot U_{\infty }, \end{array}$$

where *U*_∞_ is the neuron's steady-state membrane depolarization (as in Equation 3), if the spiking mechanism (Equation 10) is disabled. We will refer to *U*_∞_ as the “target voltage.” When the spiking mechanism is enabled, then steady-state spiking occurs if *U*_∞_ > θ_∞_, which we show below.

The steady-state spiking frequency of a neuron *f*_*sp*_ is the inverse of the time required for the neuron's membrane potential in Equation (5) to cross the threshold θ(*t*),

(13) $$\begin{array}{l} f_{sp}=\frac{-1}{\tau_{mem}\cdot \text{ln}\left(1-\frac{\theta }{U_{\infty }}\right)}. \end{array}$$

Equation (13) indicates that if the target voltage is below the threshold (i.e., *U*_∞_ < θ), then the argument of ln() becomes <0 and the frequency is undefined because no spikes can occur. Increasing the target voltage *U*_∞_ (e.g., via synaptic inputs or applied current) or decreasing the threshold θ increases *f*_*sp*_ by decreasing the amount of time needed for *U*(*t*) to reach θ. The spiking frequency can also be increased by reducing τ_*mem*_, which similarly reduces the time needed for *U*(*t*) to reach θ.

If the spiking threshold *is not* dependent on the membrane voltage (i.e., *m* = 0 in Equation 10), then θ(*t*) = θ_0_, and Equation (13) is used to calculate the spiking frequency explicitly, given the target voltage *U*_∞_. According to Equation (3), *U*_∞_ is a function of *I*_*app*_, so Equation (13) provides the *I*_*app*_ → *f*_*sp*_ mapping necessary to demonstrate parallel P1 when *m* = 0.

The spiking threshold may depend on the membrane potential (*m* ≠ 0 in Equation 10), for example, when modeling neurons that exhibit class 2 excitability, frequency adaptation, or other non-constant spiking frequency responses (Mihalaş and Niebur, 2009). In such cases, Equation (13) is still used to calculate the spiking frequency. However, since θ is a dynamical variable, the value of θ at the instant that a spike occurs, called θ^*^ must be found. Additionally, θ^*^ may change from spike to spike, so in fact one must solve for $\theta_{\infty }^{*}$, the instantaneous spiking threshold for each spike during steady-state spiking. This amounts to solving the following implicit equation, which is derived in the Supplementary Materials S1.1.1 and S1.1.2:

(14) $$\begin{array}{l} f\left(\theta_{\infty }^{*}\right)=0=\\ \{\begin{array}{cc} \;(\theta_{\infty }-\theta_{\infty }^{*})\cdot \frac{\theta_{\infty }^{*}}{U_{\infty }}+m\cdot U_{\infty }\cdot \left(1-\frac{\theta_{\infty }^{*}}{U_{\infty }}\right)\cdot \text{ln}\left(1-\frac{\theta_{\infty }^{*}}{U_{\infty }}\right),\\ \text{ if }\tau_{mem}=\tau_{\theta };\left(\theta_{\infty }-\theta_{\infty }^{*}\right)\cdot \left(1-{\left(1-\frac{\theta_{\infty }^{*}}{U_{\infty }}\right)}^{\tau_{mem}/\tau_{\theta }}\right)+\\ \;\frac{m\cdot U_{\infty }\cdot \tau_{mem}}{\tau_{\theta }-\tau_{mem}}\cdot \left(\left(1-\frac{\theta_{\infty }^{*}}{U_{\infty }}\right)-{\left(1-\frac{\theta_{\infty }^{*}}{U_{\infty }}\right)}^{\tau_{mem}/\tau_{\theta }}\right), & \text{else}. \end{array} \end{array}$$

Equation (14) implicitly describes the relationship between the target voltage *U*_∞_ and the threshold at the instant a spike occurs $\theta_{\infty }^{*}$, and must be solved numerically. Once $\theta_{\infty }^{*}$ is found, the steady-state spiking frequency is calculated by substituting $\theta =\theta_{\infty }^{*}$ in Equation (13).

A more easily computed but less accurate explicit function approximation of $\theta_{\infty }^{*}$ based on *U*_∞_ is shown below in Equation (16). This approximation follows from observing that when *f*_*sp*_ is large, two key phenomena arise: First, there is less time between spikes for θ to fluctuate, so its average value is a good estimate of its instantaneous value; second, the average voltage *U*_*avg*_ approaches $\theta_{\infty }^{*}/2$ (Supplementary Materials S1.1.3), enabling the usage of Equation (12) to calculate

(15) $$\begin{array}{l} \theta_{\infty }^{*}=\theta_{0}+m\cdot \frac{\theta_{\infty }^{*}}{2}. \end{array}$$

Rearranging for $\theta_{\infty }^{*}$ yields

(16) $$\begin{array}{l} \theta_{\infty }^{*}=\frac{\theta_{0}}{1-m/2}=B\cdot \theta_{0}, \end{array}$$

where *B* = 1/(1 − *m*/2). The approximation in Equation (16) has the advantage of being explicit. However, it is only accurate when 1/*f*_*sp*_ << τ_θ_. Its utility will be explored in the results (section 3).

#### 2.2.1. Average Synaptic Conductance

The average synaptic conductance *G*_*avg*_ is computed by solving for *G*_*s*_(*t*) and calculating its average value over a duration of one interspike period, *T*_*sp*_ = 1/*f*_*sp*_. The synaptic conductance evolves according to Equation (11). Because a pre-synaptic spike resets the conductance to *G*_*max*_, the conductance after a spike at time *t* = 0 is simply

(17) $$\begin{array}{l} G_{s}\left(t\right)=G_{max}\cdot e^{-t/\tau_{s}}. \end{array}$$

The average conductance *G*_*avg*_ given a steady-state pre-synaptic interspike period *T*_*sp*_ can be calculated by integrating Equation (17) over the interval [0, *T*_*sp*_] and dividing by *T*_*sp*_. Performing this integral,

(18) $$\begin{array}{l} G_{avg}=G_{max}\cdot \tau_{s}\cdot f_{sp}\cdot \left(1-e^{-1/f_{sp}\cdot \tau_{s}}\right). \end{array}$$

The average conductance *G*_*avg*_ is directly proportional to the pre-synaptic spiking frequency *f*_*sp*_ except for the influence of the exponential term, which we define as

(19) $$\begin{array}{l} \delta =e^{-1/f_{sp}\cdot \tau_{s}} \end{array}$$

This equation will be necessary to tune synaptic connections in the network.

### 2.3. Network Construction

To control motion, the nervous system must map from mechanical quantities (e.g., muscle stretch) to neural quantities (e.g., sensory neuron voltage, spiking frequency, etc.), perform computations, and then map neural output back into mechanical quantities (e.g., muscle force) (Eliasmith and Anderson, 2003; Szczecinski et al., 2017b; Hilts et al., 2019). Thus, the network “encodes” the mechanical state of the animal or robot, performs control computations, and then “decodes” the required actuator forces. For example, Figure 1 illustrates a simple stretch reflex, implemented as both non-spiking and spiking pathways. The type of encoding and decoding that take place in each pathway depends on whether it is spiking or non-spiking, but the computation itself should not differ.

In this manuscript, each non-spiking neuron has a maximum expected membrane depolarization *R*, and each spiking neuron has a maximum expected spiking frequency *F*_*max*_. The minimum value in each case is 0. Specifying an expected range of activity for each type of network simplifies network tuning in two ways. First, it enables a clear comparison between non-spiking and spiking implementations of the same network. The activation of analogous nodes in two networks should be equal once normalized to the range of activity, e.g., $\bar{U}$/*R* = *f*_*sp*_/*F*_*max*_. When all of a network's nodes have the same activity scale, more specific and precise computations can be designed within the network (Szczecinski et al., 2017b). Second, it enables synaptic connections between serial nodes in the same network to be parameterized by the gain *k*_*syn*_, whether non-spiking or spiking models are used. The gain can be formulated as either *k*_*syn*_ = $\bar{U}$_*post*_/$\bar{U}$_*pre*_ or *k*_*syn*_ = *f*_*post*_/*f*_*pre*_. Normalizing network activity in this way will enable us to apply the same design constraints derived in Szczecinski et al. (2017b) to the spiking model.

### 2.4. Simulation

All simulations were implemented in Matlab (The Mathworks, Natick, MA). Neurons were initialized with a random initial depolarization *U*(0) ∈ [0, θ_0_]. This added some variation to the simulation. All units were scaled to ms, mV, nA, nF, and μS (MΩ). Dynamics were simulated using the forward Euler method, with time step $\Delta t=\text{min}\left(\tau_{mem}\right)\cdot 10^{-4}$ ms.

## 3. Results

### 3.1. Comparison of Non-spiking and Spiking Neuron Activation

The steady-state spiking frequency *f*_*sp*_ is approximately a linear function of the applied current *I*_*app*_. Equation (4) shows that the non-spiking model's voltage $\bar{U}$ is directly proportional to *I*_*app*_. However, Equation (13) shows that the spiking frequency of the spiking model *f*_*sp*_ is a transcendental function of the neuron's applied current *I*_*app*_. Despite its transcendental nature, it can be bounded by parallel lines,

(20) $$\begin{array}{l} \frac{I_{app}}{G_{mem}\cdot \tau_{mem}\cdot \theta_{\infty }^{*}}-\frac{1}{2\cdot \tau_{mem}}\leq f_{sp}\left(I_{app},\theta_{\infty }^{*}\right)\\ \begin{array}{l} \;\leq \frac{I_{app}}{G_{mem}\cdot \tau_{mem}\cdot \theta_{\infty }^{*}}+\frac{1}{2\cdot \tau_{mem}}, \end{array} \end{array}$$

provided that

(21) $$\begin{array}{l} I_{bias}=\frac{G_{m}\cdot \theta_{\infty }^{*}}{2}. \end{array}$$

**Figure 2:**
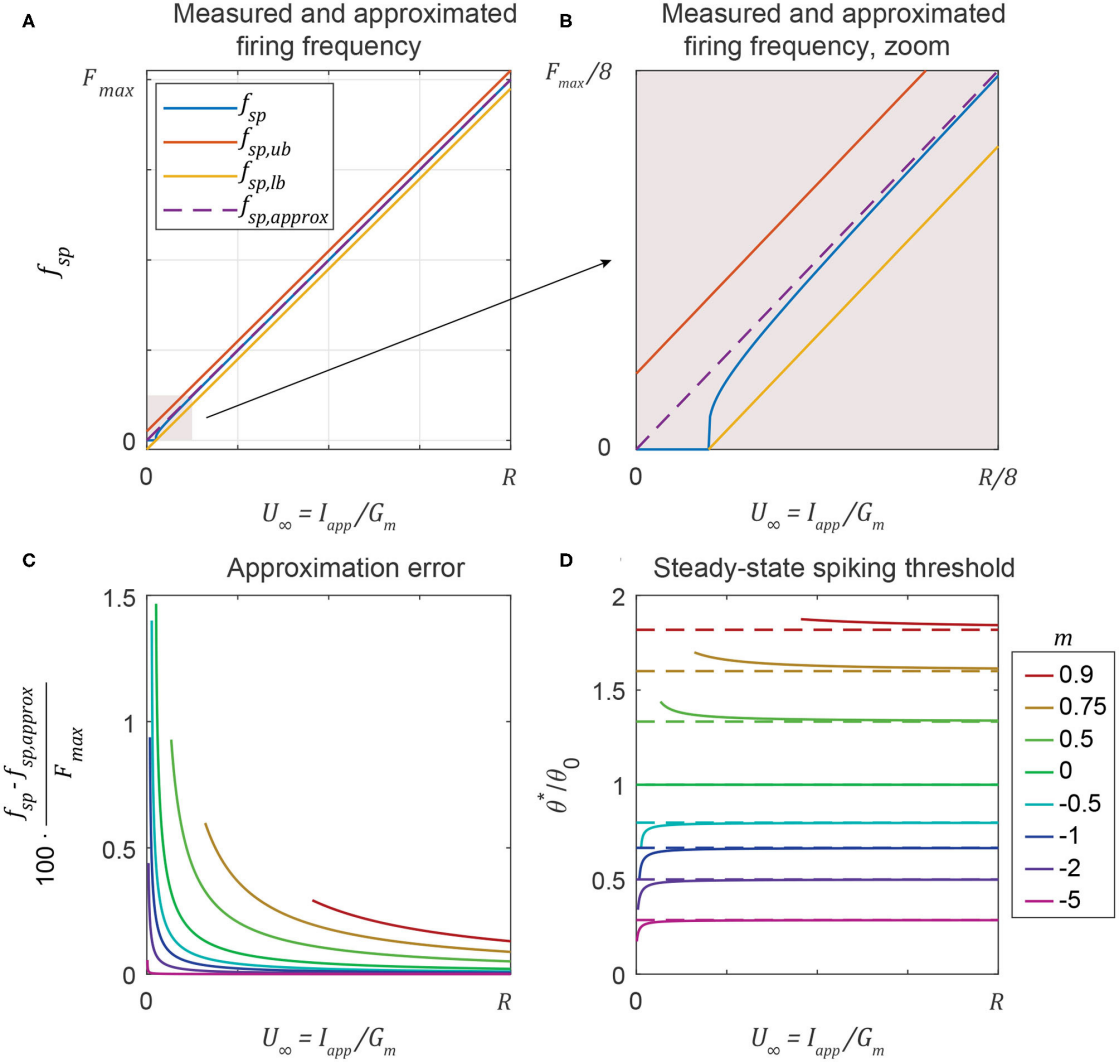
**(A)** A neuron's spiking frequency *f*_*sp*_ is an approximately linear function of the steady-state membrane voltage *U*_∞_, staying between the upper bound $f_{sp,ub}=\left(U_{\infty }+\theta_{\infty }^{*}/2\right)/\left(\tau_{mem}\cdot \theta_{\infty }^{*}\right)$ and the lower bound $f_{sp,lb}=\left(U_{\infty }-\theta_{\infty }^{*}/2\right)/\left(\tau_{mem}\cdot \theta_{\infty }^{*}\right)$ (Equation 20). **(B)** Zooming in shows that the linear approximation is poor at low frequencies. **(C)** The error between the measured and approximated spiking frequency is a function of both *U*_∞_ and *m*, but stays below 2% in all cases. **(D)** Even if the spiking threshold can change over time (*m* ≠ 0), neurons tend to spike at the same threshold no matter the value of *U*_∞_. This threshold approaches the explicit approximation in Equation (16) (dashed lines) as *U*_∞_ increases.

This inequality is derived in Supplementary Materials (S1.1.4). For any *I*_*app*_, the precise value of $\theta_{\infty }^{*}$ is calculated numerically with Equation (14), or approximated with the explicit function in Equation (16). Then, Equation (20) provides affine bounds on the spiking frequency, with a range of ±1/(2·τ_*mem*_). Figures 2A,B plot the three terms in Equation (20) vs. *I*_*app*_/*G*_*mem*_. Figure 2A shows that as *I*_*app*_ increases, *f*_*sp*_ is an approximately linear function of *I*_*app*_, and approaches the mean of the bounds from Equation (20). The resulting approximation is

(22) $$\begin{array}{l} f_{sp,approx}\left(I_{app},\theta_{\infty }^{*}\right)=\frac{I_{app}}{G_{mem}\cdot \tau_{mem}\cdot \theta_{\infty }^{*}}. \end{array}$$

Figure 2B shows that the linear approximation in Equation (22) breaks down for very small values of *I*_*app*_, but Figure 2C shows that the error asymptotically approaches 0 as *I*_*app*_ increases, no matter the value of *m*. Figure 2D shows that for any value of *m*, $\theta_{\infty }^{*}$ approaches the value in Equation (16) as *I*_*app*_ (and *f*_*sp*_) increases.

Equation (22) reveals the parallel between the non-spiking activation $\bar{U}$ and the spiking activation *f*_*sp*_: Both are directly proportional to *I*_*app*_ (provided the bias current is tuned according to Equation 21). To strengthen this parallel, τ_*mem*_ can be tuned such that each activation value equals its maximum value when the input *I*_*app*_ equals its maximum value, *G*_*mem*_ · *R*. The resulting constraint is

(23) $$\begin{array}{l} \tau_{mem}=\frac{R}{F_{max}\cdot \theta_{\infty }^{*}}. \end{array}$$

This condition implies that given the same input current *I*_*app*_, the non-spiking and spiking activations can be mapped to each other through the relationship

(24) $$\begin{array}{l} f_{sp,approx}\left(I_{app},\theta_{\infty }^{*}\right)=\frac{F_{max}}{R}\cdot {\bar{U}}_{\infty }, \end{array}$$

The results show that the spiking frequency is parallel to the membrane depolarization of the non-spiking model. Each activation state is a linear function of the applied current, and can be related to each other by the factor *F*_*max*_/*R*. This supports parallel P1 from the Introduction: Non-spiking neuron voltage is analogous to the spiking neuron's spiking frequency.

### 3.2. Comparison of Non-spiking and Spiking Activation Transient Responses

The transient spiking threshold θ^*^ can be tuned to approximate the transient response of the non-spiking membrane depolarization $\bar{U}$. Knowing the steady-state spiking threshold at the time of spiking, $\theta_{\infty }^{*}$, one can calculate the evolution of θ^*^ from spike to spike. This evolution describes the transient response of the neuron's spiking frequency in response to a step-input current. The following expression is derived in Appendix 1.4:

(25) $$\begin{array}{l} \theta^{*}\left(t\right)=\theta_{\infty }^{*}+\left(\theta_{0}-\theta_{\infty }^{*}\right)\cdot e^{-t/\tau_{\theta^{*}}}, \end{array}$$

where $\theta_{\infty }^{*}$ is calculated via Equation (14) or Equation (16) and $\tau_{\theta^{*}}=\tau_{\theta }\cdot B$ (Equation 46).

Equation 25 describes how the spiking threshold, and thus the spiking frequency, evolves from spike time to spike time. This is analogous to the non-spiking neuron membrane transient response from Equation (5), whose time constant is defined in Equation (6). The time constants in a non-spiking functional subnetwork (e.g., as designed according to Szczecinski et al., 2017b) are mapped to the threshold's time constant in a spiking neuron network with the equation

(26) $$\begin{array}{l} \tau_{\theta }={\overset{-}{\tau }}_{mem}\cdot \left(1-\frac{m}{2}\right). \end{array}$$

**Figure 3:**
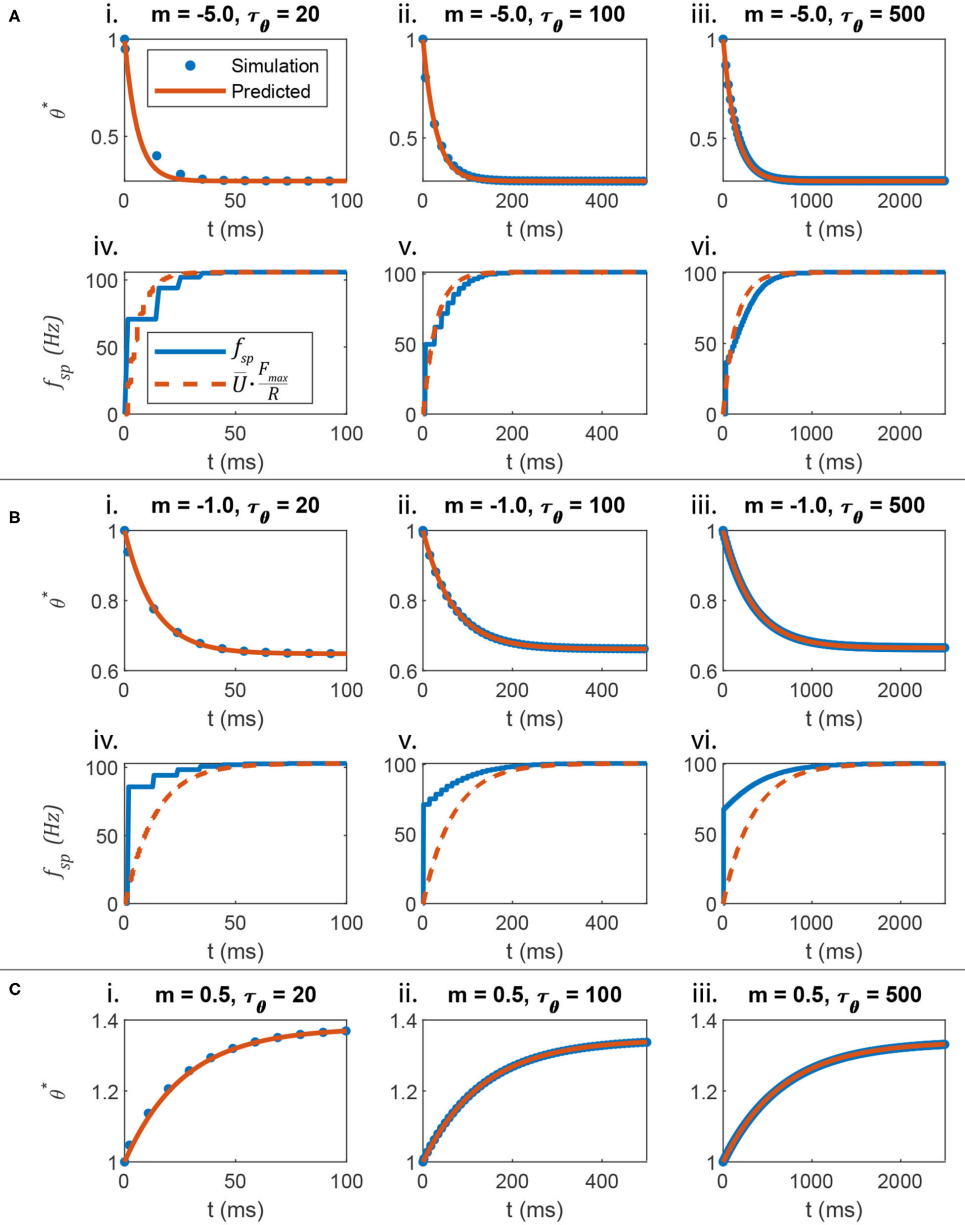
The evolution of the spiking threshold when a spike occurs can be predicted. **(A)** When the threshold strongly hyperpolarizes in response to membrane voltage depolarization, the transient spiking frequency evolves much like the non-spiking neuron's membrane voltage. **(B)** When the threshold weakly hyperpolarizes in response to membrane voltage depolarization, the transient spiking frequency evolves at the same rate as a non-spiking neuron's membrane voltage, but the amplitude matches poorly. This is because the spiking neuron's membrane must depolarize strongly before firing even one spike, leading to a large leap in frequency from 0 to a non-zero value. **(C)** This analysis also applies if the spiking threshold depolarizes in response to membrane voltage depolarization (i.e., *m* > 0). However, the comparison between *f*_*sp*_ and *U* is not shown because in this case, *f*_*sp*_ begins large and decreases over time, rather than beginning small and increasing over time, like $\bar{U}$.

Figure 3A plots the response of θ and *f*_*sp*_ when *m* = −5, compared to the response of a non-spiking neuron voltage $\bar{U}$, scaled by *F*_*max*_/*R* (Equation 24). Figures 3Ai–iii show that Equation (25) accurately predicts the spiking threshold at each spike time, even as different values of τ_θ_ are used. Figures 3Aiv–vi show that when *m* = −5, *f*_*sp*_ evolves smoothly, because θ^*^ evolves quickly and with a large amplitude.

Figure 3B plots the transient responses when *m* = −0.5. θ^*^ is predicted accurately in Figures 3Bi–iii. However, the spiking frequency discontinuously leaps from 0 to a non-zero value as shown in Figures 3Biv–vi. Since θ has the same transient time constant as $\bar{U}$, the transient firing frequency has the same decay rate as the non-spiking neuron voltage. However, the amplitude does not map directly.

Finally, Figures 3Ci–iii show that this analysis applies even when *m* > 0, that is, when the threshold *increases* as *U* increases. Figure 3C does not include plots of *f*_*sp*_ or $\bar{U}$ because when *m* > 0, *f*_*sp*_ *decreases* over time, a behavior that the non-spiking model cannot reproduce.

The data in Figure 3A show that when *m* << 0, the firing frequency *f*_*sp*_ evolves smoothly at the same rate as $\bar{U}$ scaled by *F*_*max*_/*R*, when subjected to the same current input. This result supports parallel P2 from the Introduction: The transient spiking threshold θ^*^ mimics the transient membrane depolarization $\bar{U}$ as long as $\tau_{\theta^{*}}=\tau_{\theta }\cdot B={\overset{-}{\tau }}_{mem}$.

### 3.3. Comparison of Non-spiking and Spiking Synaptic Conductance

**Figure 4:**
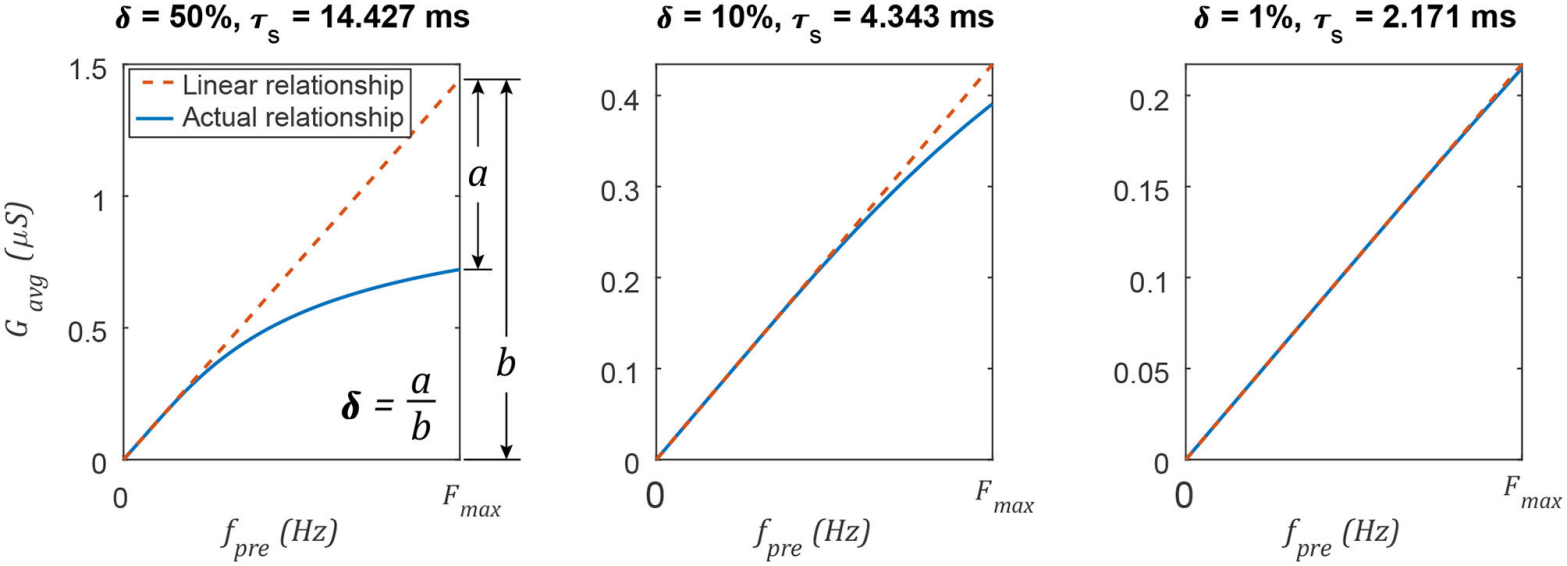
Decreasing the synaptic time constant τ_*s*_ increases the linearity of the average synaptic conductance *G*_*avg*_ as a function of the pre-synaptic neuron's spiking frequency *f*_*pre*_. Changing the network's maximum spiking frequency *F*_*max*_ will change the specific value of τ_*s*_, but the linearity δ varies in the same way.

The average spiking synaptic conductance *G*_*avg*_ can be tuned to approximate the non-spiking synaptic conductance $\bar{G}$_*s*_. *G*_*avg*_ is approximately proportional to *f*_*sp*_ when τ_*s*_ is sufficiently small. Decreasing τ_*s*_ improves this approximation by reducing the impact of δ, the exponential term in Equation (18). Figure 4 illustrates δ graphically. After selecting a value for δ, Equation (19) can be solved to compute the upper limit of τ_*s*_,

(27) $$\begin{array}{l} \tau_{s}\leq \frac{-1}{F_{max}\cdot \text{ln }\delta }\;. \end{array}$$

For example, if δ = 0.01 (1% deviation from a linear relationship) and *F*_*max*_ = 0.1 kHz, then τ_*s*_ ≤ 2.17 ms. Equation 27 is necessary to ensure that the average synaptic conductance *G*_*avg*_ is an approximately linear function of the pre-synaptic neuron's firing frequency *f*_*sp*_.

Figure 4 plots *G*_*avg*_ vs. *f*_*sp*_ for various parameter value combinations. δ is different in each column. In each case, τ_*s*_ is calculated using Equation (27). When δ is small (≤ 1%), the average spiking synaptic conductance is almost directly proportional to the spiking frequency of the pre-synaptic neuron. This is analogous to the non-spiking synaptic conductance, which is proportional to the membrane depolarization of the pre-synaptic neuron (Equation 2). This result supports parallel P3 from the Introduction: The average spiking synaptic conductance *G*_*avg*_ is proportional to the pre-synaptic neuron's activation. However, *G*_*avg*_ is an emergent property of the synapse; the designer can only set the value of *G*_*max*_. How should *G*_*max*_ be set? Once the desired value for *G*_*avg*_ is known (see section 3.4), *G*_*max*_ is determined by rearranging Equation (18),

(28) $$\begin{array}{l} G_{max}=\frac{G_{avg}}{\tau_{s}\cdot F_{max}\cdot \left(1-\delta \right)}. \end{array}$$

What if one wishes to set *G*_*max*_ to achieve a particular functional gain, that is, ratio of firing frequencies between the post-synaptic and pre-synaptic neurons (i.e., *f*_*sp,post*_/*f*_*sp,pre*_)? In this case, one sets *G*_*avg*_ in Equation (28) equal to the equivalent non-spiking synaptic conductance in Equation (7), which is expressed in terms of *k*_*syn*_, the functional synaptic gain. As a further simplification, let us assume δ ≈ 0, in which case:

(29) $$\begin{array}{l} G_{max}=\frac{k_{syn}\cdot R}{\left(E-k_{syn}\cdot R\right)\cdot \tau_{s}\cdot F_{max}}. \end{array}$$

This equation enables the direct design of synaptic conductance based on synaptic gain.

### 3.4. Extending the Functional Subnetwork Approach for Designing Non-spiking Networks to Spiking Networks

Having established analogous parameters between non-spiking and spiking networks, we can now adapt the functional subnetwork approach (FSA) (Szczecinski et al., 2017b) to spiking networks. The goal of the FSA is to determine the parameter values for individual neurons and synapses based on network-wide parameter values (e.g., *R* and *F*_*max*_) and the intended function of a portion of a network. In the non-spiking framework we previously developed, one could construct networks that could add two (or *N*) input signals using three (or *N*+1) neurons; subtract two signals using three neurons; multiply two signals using four neurons; divide two signals using three neurons; differentiate a signal using four neurons; and integrate a signal over time using two neurons. The accuracy of the FSA has a practical limit, especially when parameter values are constrained to biologically plausible bounds. However, the FSA enables the rapid, direct assembly of networks that can perform sophisticated tasks, such as entraining rhythmic output to periodic inputs (Nourse et al., 2018) or learning the appropriate force to apply to the environment (Szczecinski and Quinn, 2017a), right “out of the box.” Such networks also serve as good starting configurations for subsequent optimization (Pickard et al., 2020). As such, we find the FSA to be useful for designing computational models and robot controllers.

How does one use the FSA to select parameter values based on network function? As an example, consider a pathway in which one neuron's activation may represent the average of two other neurons' activation. To accomplish this, the synapses connecting these neurons should each have gain *k*_*syn*_ = 1/2, such that *f*_3_ = 1/2 · (*f*_1_ + *f*_2_). For another example, one neuron's activation may represent the difference between two other neurons' activation if the synapses have equal and opposite gain, e.g., *k*_*syn*,1_ = 1, *k*_*syn*,2_ = −1 (and the second synapse has a negative reversal potential). The FSA ensures that each neuron encodes the intended quantities and performs the intended operation by tuning parameter values in an algorithmic way.

Table 2 contains the FSA for spiking networks. The designer first sets network-wide values for the spiking neuron parameters *F*_*max*_, θ_0_, *R*, either based on biological data or arbitrarily. Applying the same values across the entire network ensures proper encoding and decoding (e.g., Figure 1). Next, the designer sets *m* and τ_θ_ based on the desired transients in the system. Then *I*_*bias*_ and τ_*mem*_ are calculated so that *f*_*sp*_ = *F*_*max*_ when *I*_*app*_/*G*_*m*_ = *R*. After the neural parameters, the synaptic parameters are tuned. The synaptic decay constant τ_*s*_ is calculated to ensure the average synaptic conductance is proportional to the pre-synaptic neuron's spiking frequency. Finally, the maximum synaptic conductance *G*_*max*_ is calculated to achieve the intended average synaptic conductance.

**Table 2:** Expanded functional subnetwork approach for designing spiking pathways.

**Step**	**Goal**	**Param**.	**Eq. No**.	**Equation**
1.	Set network-wide activation parameters.	*F*_*max*_, *R*, and θ_0_	24	$f_{sp,approx}=\frac{F_{max}}{R}\cdot {\bar{U}}_{\infty }$
2.	For each neuron, set transient response type based on function in the network.	*m*		For $\frac{\text{d}}{\text{dt}}f_{sp}>0$, set *m* < 0. For $\frac{\text{d}}{\text{dt}}f_{sp}=0$, set *m* = 0. For $\frac{\text{d}}{\text{dt}}f_{sp}<0$, set *m* > 0.
3.	For each neuron, set the duration of transient firing based on its function in the network.	τ_θ_	26	$\tau_{\theta }={\overset{-}{\tau }}_{mem}\cdot \left(1-\frac{m}{2}\right)$
4.	For each neuron, calculate bias current.	*I*_*bias*_	21	$I_{bias}=\frac{G_{mem}\cdot \theta_{0}}{2-m}$
5.	For each neuron, ensure *f*_*sp*_ ∈ [0, *F*_*max*_].	τ_*mem*_	23	$\tau_{mem}=\frac{R}{F_{max}}\cdot \frac{1-\frac{m}{2}}{\theta_{0}}$
6.	For each synapse, limit its non-linearity.	τ_*s*_	27	$\tau_{s}\leq \frac{-1}{F_{max}\cdot \text{ln}\delta }$
7.	For each synapse, set synaptic gain *k*_*syn*_ based on function in the network.	*G*_*max*_	29	$G_{max}=\frac{k_{syn}\cdot R}{\left(E_{s}-k_{syn}\cdot R\right)\cdot \tau_{s}\cdot F_{max}}$

As an example, let us design a pathway wherein the post-synaptic neuron's spiking frequency mirrors its pre-synaptic neuron's spiking frequency. Following Table 2, the steps are:

1. Arbitrarily, but in the range of biological systems, pick the maximum spiking frequency *F*_*max*_ = 0.1 kHz, the maximum membrane depolarization *R* = 20 mV, and the initial firing threshold θ_0_ = 1 mV. These are arbitrary quantities, but may be tuned to match a specific pathway if data is available.
2. Pick *m* = 0 so θ is constant, and each neuron has no transient spiking frequency.
3. Since *m* = 0, there is no transient, and this step is skipped.
4. Calculate *I*_*bias*_ = 0.5 nA, so that *f*_*sp*_ = 0 when *I*_*app*_ = 0 and *f*_*sp*_ = *F*_*max*_ when *I*_*app*_ = *R*.
5. Using the values from steps 1 to 4, calculate τ_*mem*_ = 200 ms. The neuron properties are now set.
6. Set δ = 1% such that *G*_*avg*_ is nearly directly proportional to *f*_*sp*_ of the pre-synaptic neuron, with a maximum deviation of 1%. This condition is met if τ_*s*_ = 2.17 ms.
7. Using non-spiking network design rules from Szczecinski et al. (2017b), design a signal transmission pathway with a gain of *k*_*syn*_ = 1. For a value of *E*_*s*_ = 160, *G*_*max*_ = 0.658 μS.

**Figure 5:**
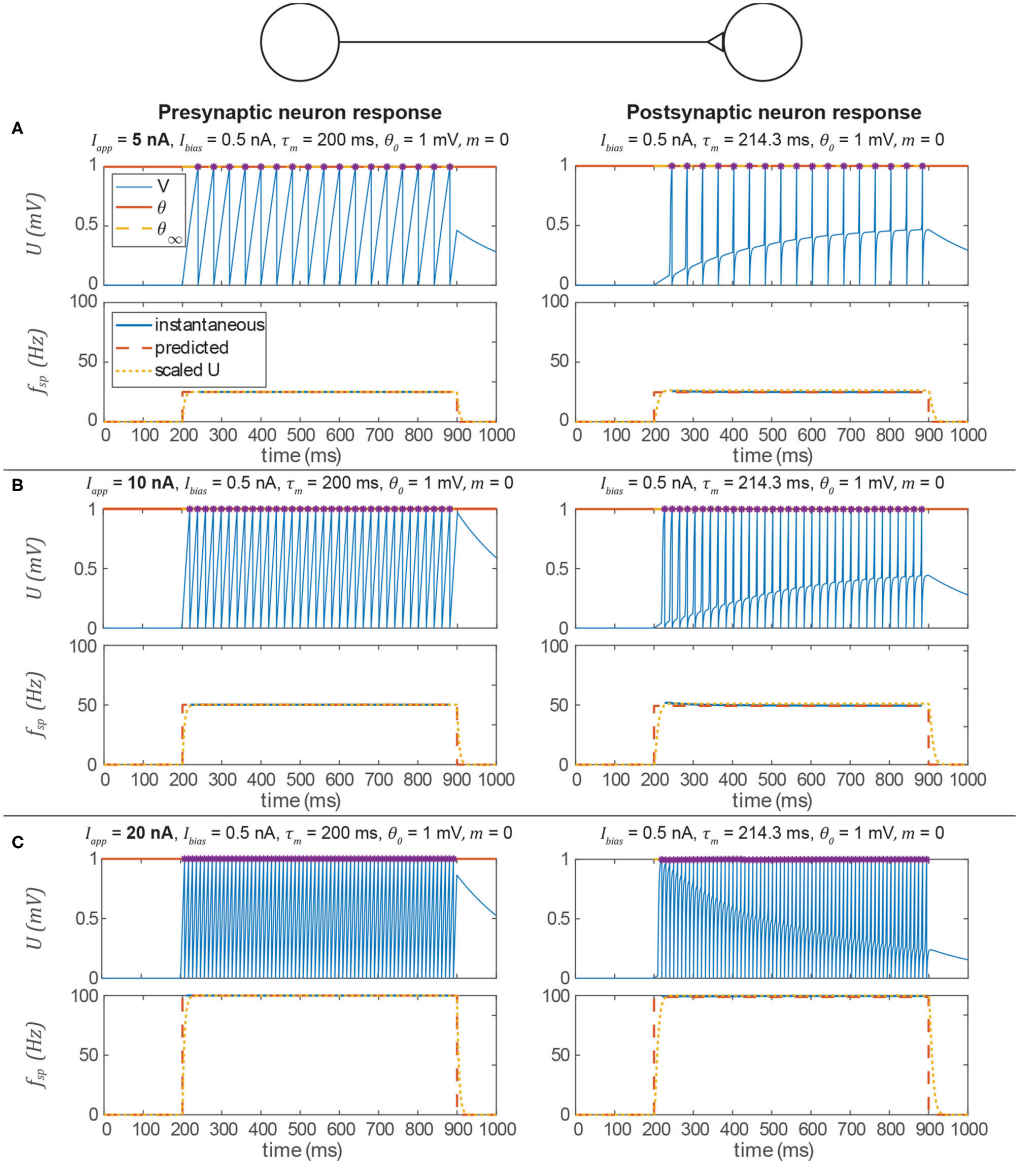
Data from three simulations testing a signal transmission pathway where *k*_*syn*_ = 1 with different applied currents (*I*_*app*_) to the pre-synaptic neuron. In each section, the upper row is the membrane voltage, and the lower row is the instantaneous spiking frequency plotted vs. time. In every case, the post-synaptic neuron's spiking frequency is the same as the pre-synaptic neuron's spiking frequency and is consistent with the prediction from the analysis above. In addition, it is approximately equal to $\bar{U}$ for the equivalent non-spiking network. Spikes are indicated by violet asterisks (no “cosmetic spikes” are plotted). **(A)** Response to a 5 nA current applied to the presynaptic neuron. **(B)** Response to a 10 nA current applied to the presynaptic neuron. **(C)** Response to a 20 nA current applied to the presynaptic neuron.

Figure 5 shows the behavior of the signal transmission pathway designed above. The left column shows the pre-synaptic neuron activity, and the right column shows the post-synaptic neuron's response. The membrane depolarization *U* is plotted in the upper row for each trial, and the spiking frequency *f*_*sp*_ is plotted in the lower row. In each case, the post-synaptic neuron's spiking frequency is effectively the same as the pre-synaptic neuron's. The key result is that the process we outline above produces parameter values for the construction of neural systems to produce a particular behavior without optimization.

Let us perform another example, in which *m* < 0, to mimic the behavior of a non-spiking neuron whose membrane time constant is ${\overset{-}{\tau }}_{mem}=500$ ms. In this case, we can also validate that the transient spiking frequency is consistent with the non-spiking model's transient membrane depolarization.

1. Pick *F*_*max*_ = 0.1 kHz, *R* = 20 mV, and θ_0_ = 1 mV.
2. Pick *m* = −5, such that the spiking frequency has a transient response.
3. If ${\overset{-}{\tau }}_{mem}=500$ ms, then τ_θ_ = 1, 750 ms.
4. Calculate *I*_*bias*_ = 0.143 nA.
5. Using the values from steps 1 to 4, calculate τ_*mem*_ = 700 ms. Steps 4 and 5 ensure that *f*_*sp*_ = 0 when *I*_*app*_ = 0 and *f*_*sp*_ = *F*_*max*_ when *I*_*app*_ = *R*. Neuron properties are now set.
6. Same as in the previous example, τ_*s*_ = 2.17 ms.
7. Same as in the previous example, *G*_*max*_ = 0.658 μS.

**Figure 6:**
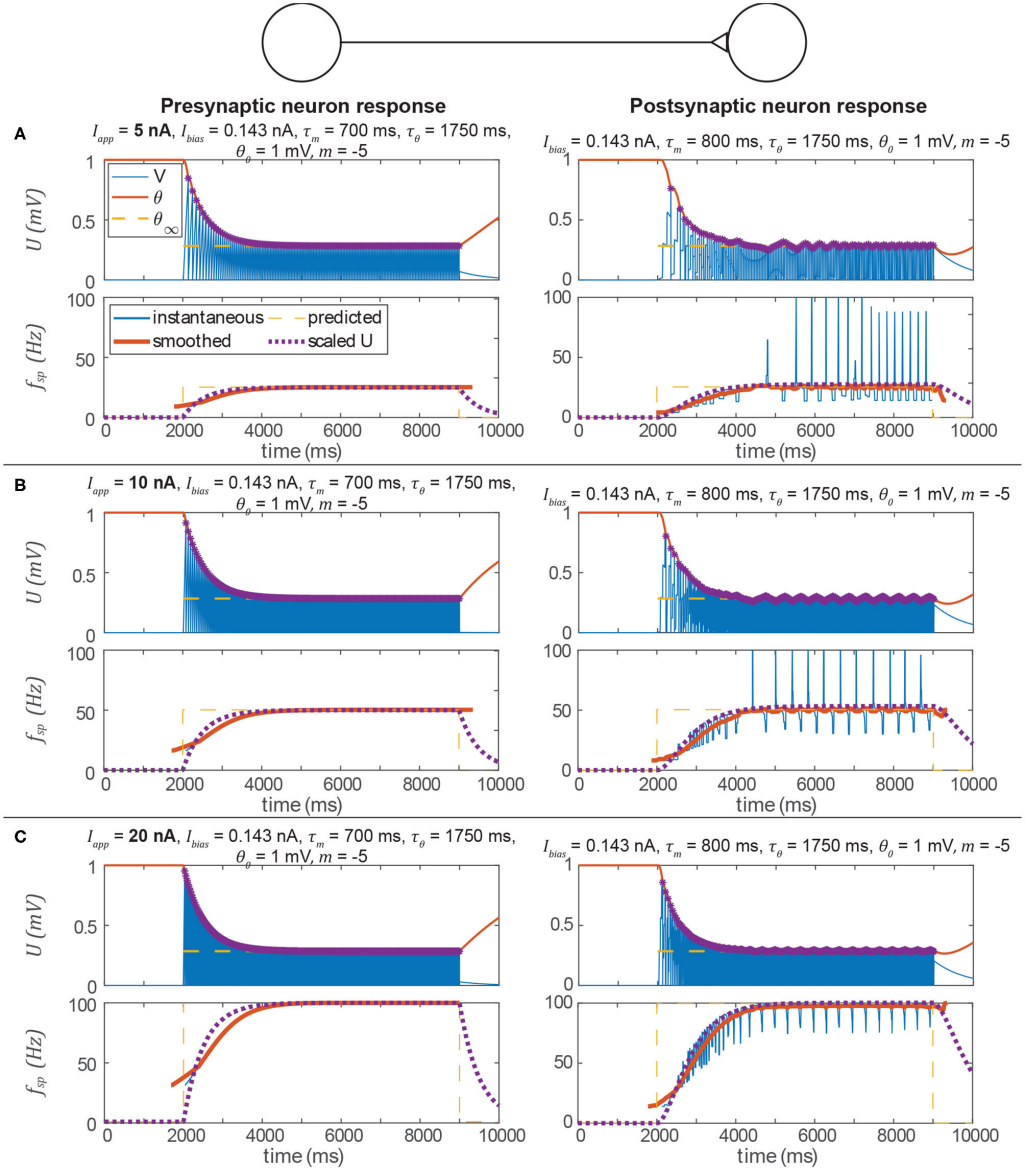
Data from three simulations testing the signal transmission pathway. In each section, the left column is the pre-synaptic neuron's activity, the right column is the post-synaptic neuron's activity, the upper row is the membrane voltage, and the lower row is the instantaneous spiking frequency (inverse of the time between two spikes) plotted vs. time. In every case, the post-synaptic neuron's spiking frequency is the same as the pre-synaptic neuron's spiking frequency, and consistent with the prediction from the analysis above. In addition, the transient spiking frequency of each neuron follows the transient membrane voltage of the analogous non-spiking pathway. Spikes are indicated by violet asterisks (no “cosmetic spikes” are plotted). **(A)** Response to a 5 nA current applied to the presynaptic neuron. Note that the presynaptic neuron's low spiking frequency causes large fluctuations in the postsynptic neuron's spiking frequency. **(B)** Response to a 10 nA current applied to the presynaptic neuron. Due to the higher spiking frequency, fluctuations in the postsynaptic neuron's spiking frequency appear less severe than in A. **(C)** Response to a 20 nA current applied to the presynaptic neuron. Fluctuations in the postsynaptic neuron's spiking frequency are not severe.

Figure 6 shows the same type of data as Figure 5. The spiking frequency plots enable us to compare the smoothed transient spiking frequency to the membrane depolarization of the analogous non-spiking network. The spiking model's smoothed transient response decays at the same rate as the non-spiking model's, although some differences in the responses are visible. First, the spiking neuron's smoothed transient does not exhibit the same exponential-shaped rise as the non-spiking neuron's transient. Second, the spiking neuron's transient response to a spiking input exhibits fluctuations because the spiking threshold is continuously adapting to the instantaneous membrane voltage. In the next subsection, we show that adding more neurons can eliminate this problem.

### 3.5. Spiking Pathways May Introduce Unwanted Artifacts

**Figure 7:**
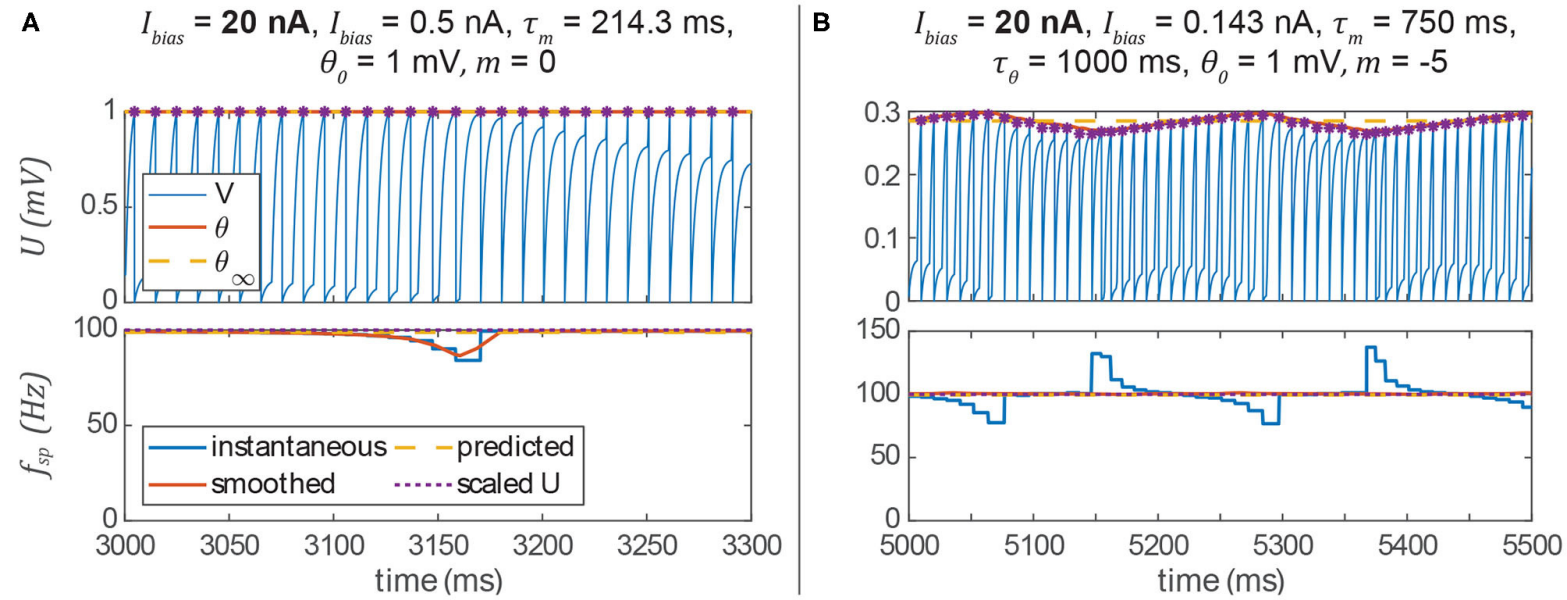
**(A)** If a pre-synaptic spike does not elicit a spike in the post-synaptic neuron, then its spiking frequency will intermittently drop. **(B)** The spiking frequency of a post-synaptic neuron whose threshold hyperpolarizes in response to membrane voltage depolarization may oscillate at low frequencies. In both columns, spikes are indicated by violet asterisks (no cosmetic “spikes” are plotted).

We have shown that we can apply our non-spiking signal pathway design rules to spiking networks by treating many values as their average over time. However, there are some unintended artifacts of this approach that reduce performance. The first is an intermittent drop in a post-synaptic neuron's spiking frequency (Figure 7A). The way the system is tuned, the post-synaptic neuron should fire every time that the pre-synaptic neuron fires. Occasionally, however, the post-synaptic spike time is delayed relative to the synaptic current. This manifests as a temporary drop in the instantaneous spiking frequency. The prediction of the average spiking frequency is intermittently incorrect as a result.

The second artifact is a periodic instantaneous spiking frequency (about the predicted spiking frequency, Figure 7B). This occurs when *m* < 0, and thus the spiking threshold θ decreases when the neuron is depolarized, making it easier for the neuron to spike. Particularly at low stimulus frequencies, the neuron may exhibit a periodic spiking frequency (Figure 7B). However, one can see that the prediction of the average spiking frequency remains accurate. In the following section, we show that both of these unwanted artifacts can be eliminated by adding more neurons and synapses to the network.

### 3.6. A Spiking Pathway's Regularity and Accuracy Depends on the Number of Neurons in the Network

**Figure 8:**
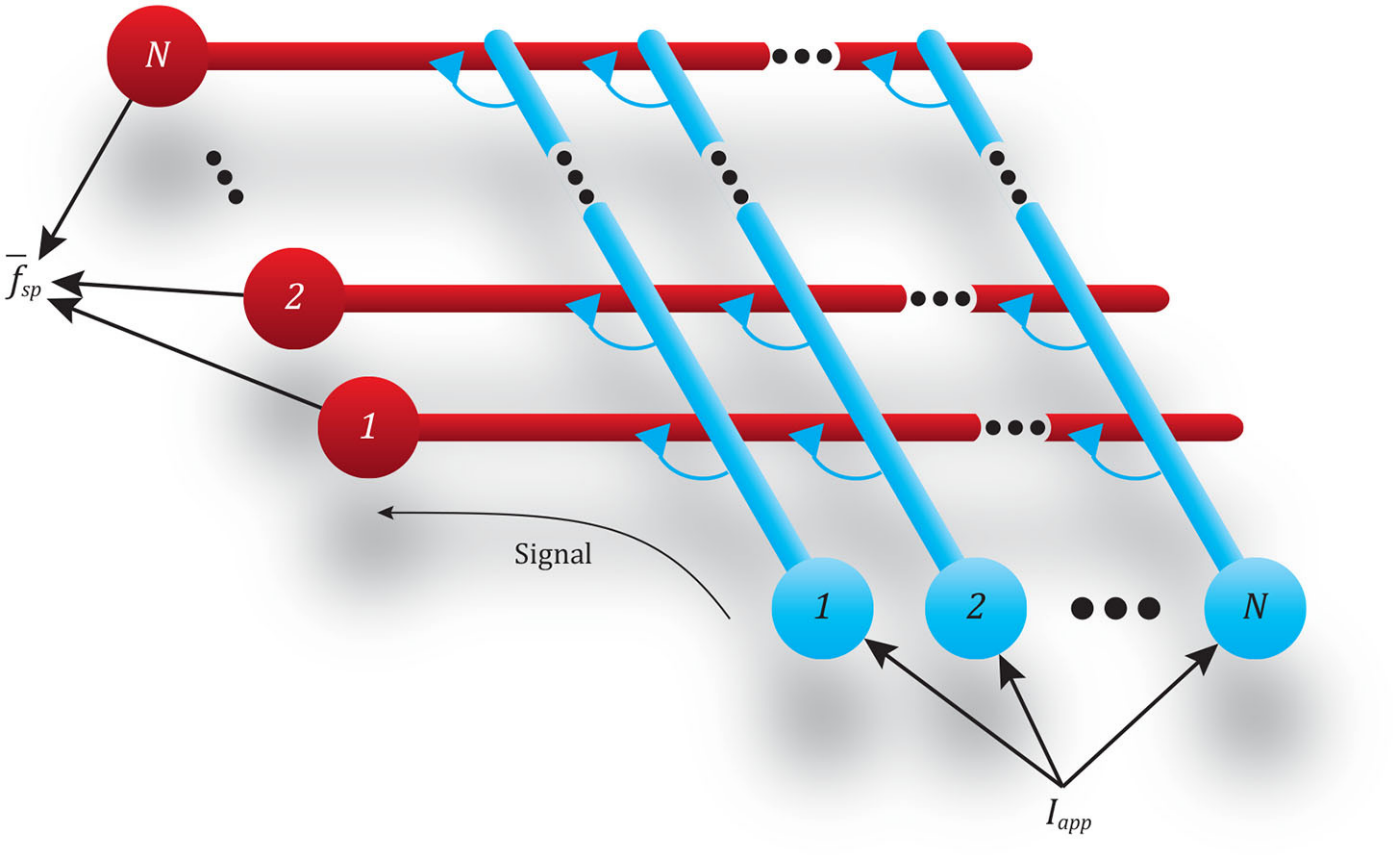
Diagram showing the all-to-all connectivity scheme used between populations of spiking neurons. The *N* input neurons (blue) all receive the same applied current *I*_*app*_, causing spikes that transmit this signal via *N*^2^ synapses to the *N* output neurons (red).

Adding more neurons to both the pre-synaptic and post-synaptic populations in a pathway helps mitigate the issues described in the previous subsection. To demonstrate this, we performed the same spiking frequency tests as before by connecting two populations of neurons together through a single pathway composed of multiple synapses. For these tests, each population has *N* neurons, and every neuron in each population is connected to every neuron in the second population, requiring *N*^2^ synapses. This connectivity pattern is illustrated in Figure 8.

**Figure 9:**
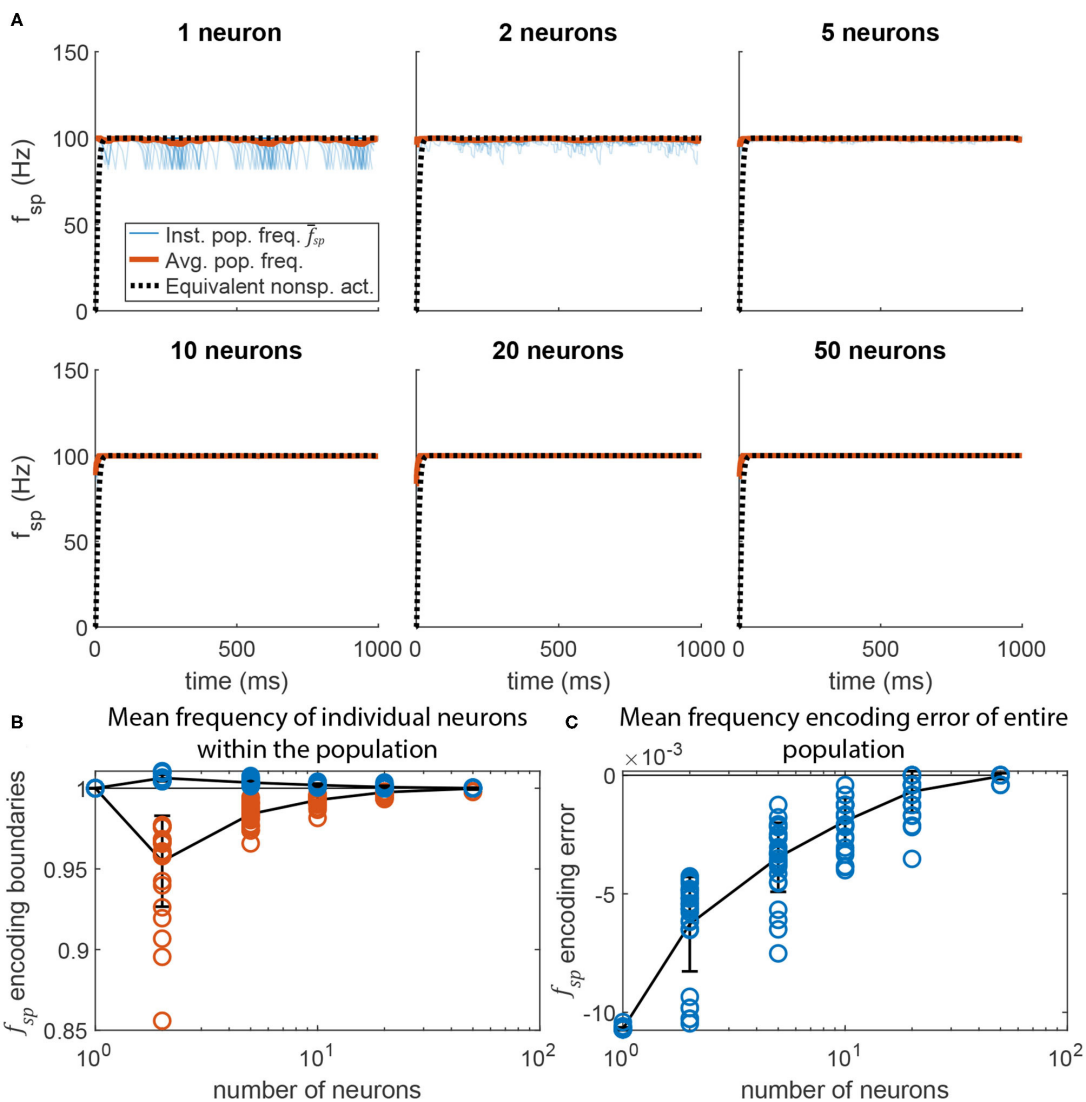
Adding more neurons to the pre-synaptic and post-synaptic populations increases encoding accuracy and reduces activation variation. **(A)** Average spiking frequency of the post-synaptic population ${\overset{-}{f}}_{sp}$ over time when the spiking threshold θ is constant (i.e., *m* = 0) for all neurons. Thirty individual trials (blue), the mean over time (red), and the equivalent non-spiking pathway (black dotted) are plotted. **(B)** The highest (blue circles) and lowest (red circles) mean frequency *of a single neuron* in the population. Line plots the mean of each group, error bars are ±1 standard deviation. **(C)** The error between the mean spiking frequency of the entire population and the intended spiking frequency for each trial (blue circles). Line plots the mean, error bars are ±1 standard deviation.

Figure 9 displays the post-synaptic population's mean spiking frequency as *N* increases. The same parameter values were used as in Figure 5, but with *G*_*max*_ randomly distributed across each neuron's *N* incoming synapses (uniform random distribution). For each value of *N*, 30 simulations were run. The raw spiking frequency over time is plotted for each. The spiking frequency fluctuates with smaller magnitude when the pathway contains more neurons. This is because each incoming synapse has a smaller maximum conductance, producing a total synaptic conductance that fluctuates less over time than when fewer synapses are present. Figure 9B plots the maximum and minimum spiking frequency of any one neuron in the population, normalized to the mean spiking frequency of the population. As *N* increases, each individual neuron's spiking frequency approaches the mean of the population. Figure 9C plots the mean spiking frequency of the population for each trial. As *N* increases, the population's spiking frequency more closely matches the intended value.

**Figure 10:**
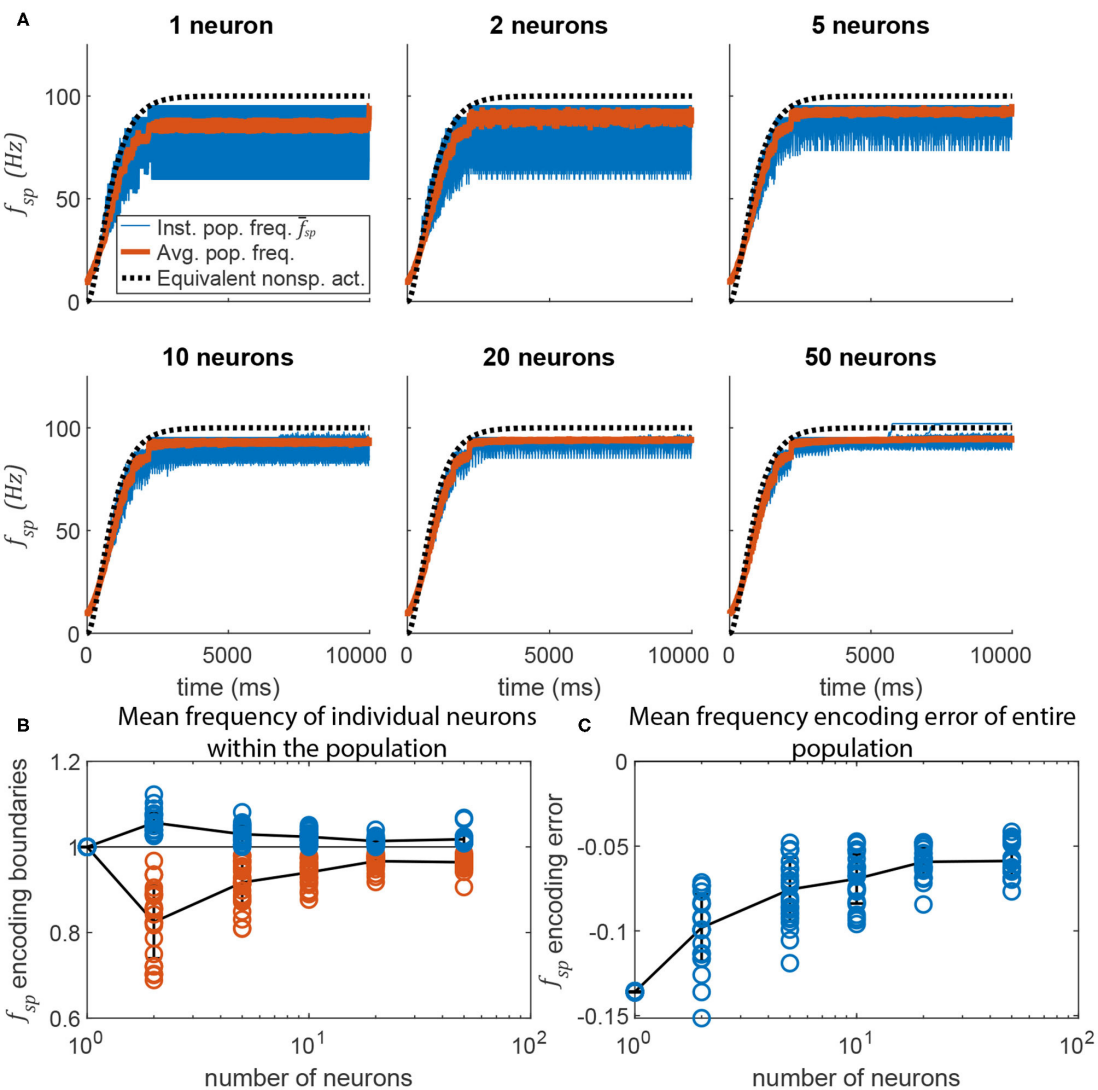
Adding more neurons to the pre-synaptic and post-synaptic populations increases encoding accuracy and reduces activation variation. **(A)** Average spiking frequency of the post-synaptic population ${\overset{-}{f}}_{sp}$ over time when the spiking threshold θ is variable (i.e., *m* = −5) for all neurons. Thirty individual trials (blue), the mean over time (red), and the equivalent non-spiking pathway (black dotted) are plotted. **(B)** The highest (blue circles) and lowest (red circles) mean frequency of a single neuron in the population. Line plots the mean, error bars are ±1 standard deviation. **(C)** The error between the mean spiking frequency of the entire population and the intended spiking frequency for each trial is plotted (blue circles). Line plots the mean, error bars are ±1 standard deviation.

Adding more neurons to a pathway also reduces the random fluctuations in the transient response of a spiking pathway in which *m* ≠ 0. Figure 10 plots data similar to that in Figure 9, but for the pathway shown in Figure 6. Much like in Figure 9, adding more neurons to the signal transmission pathway makes the post-synaptic neurons fire more regularly, and with less fluctuation during the transient. As more neurons are added, the transient response more closely matches that of the equivalent non-spiking network (in black).

## 4. Application to a Neuromechanical System

**Figure 11:**
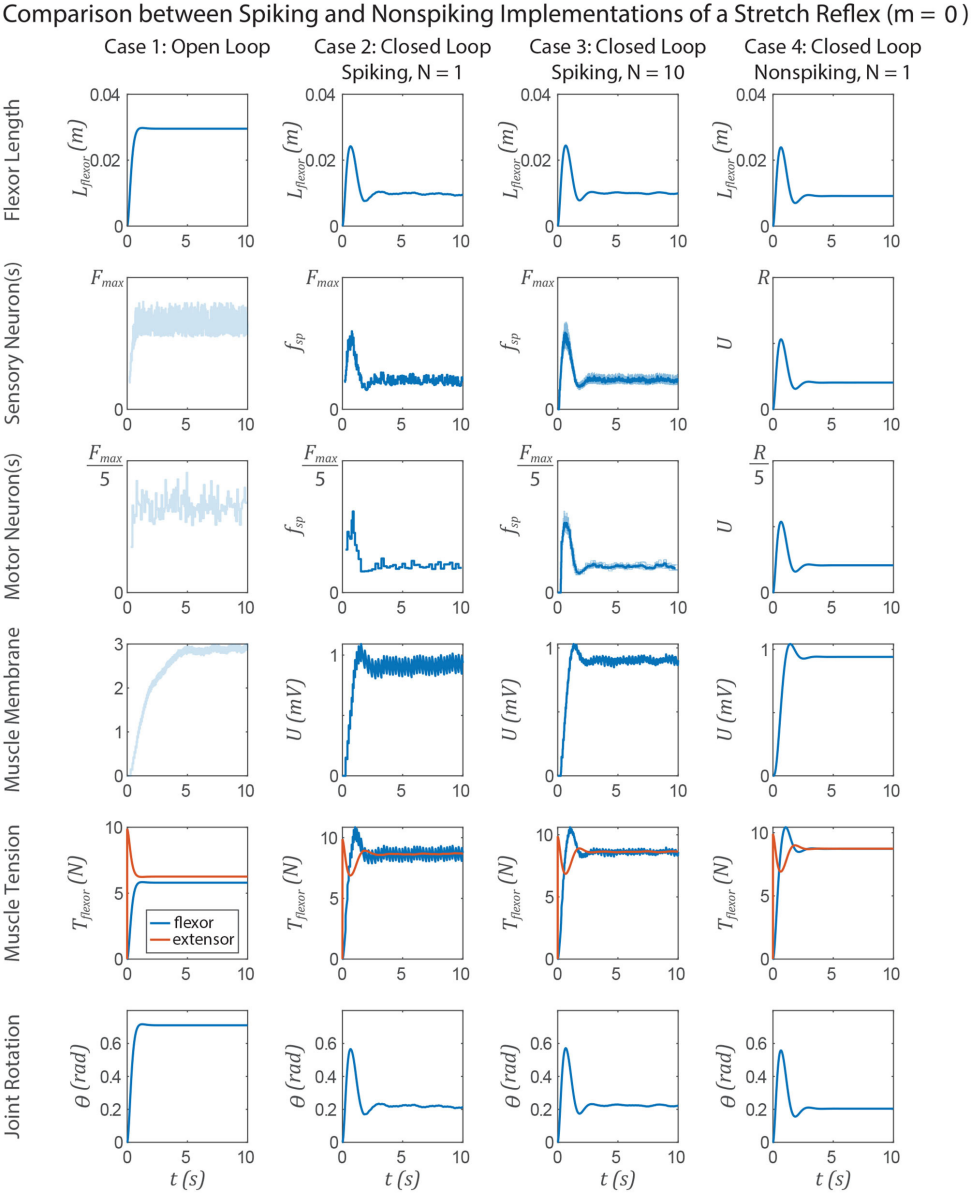
Data from a simulation of the system in Figure 1. Each column shows data from a different configuration of the system, from left to right: Case 1, open loop; case 2, spiking reflex pathway, *N* = 1; case 3, spiking reflex pathway, *N* = 10 (*f*_*sp*_ for every neuron is shown in light blue, population mean *f*_*sp*_ is plotted in dark blue); case 4, non-spiking reflex pathway. All corresponding axes are scaled the same. Each row plots data from a different stage of the reflex loop as depicted in Figure 1. In this figure, all spiking neurons have a constant spiking threshold θ (i.e., *m* = 0).

**Figure 12:**
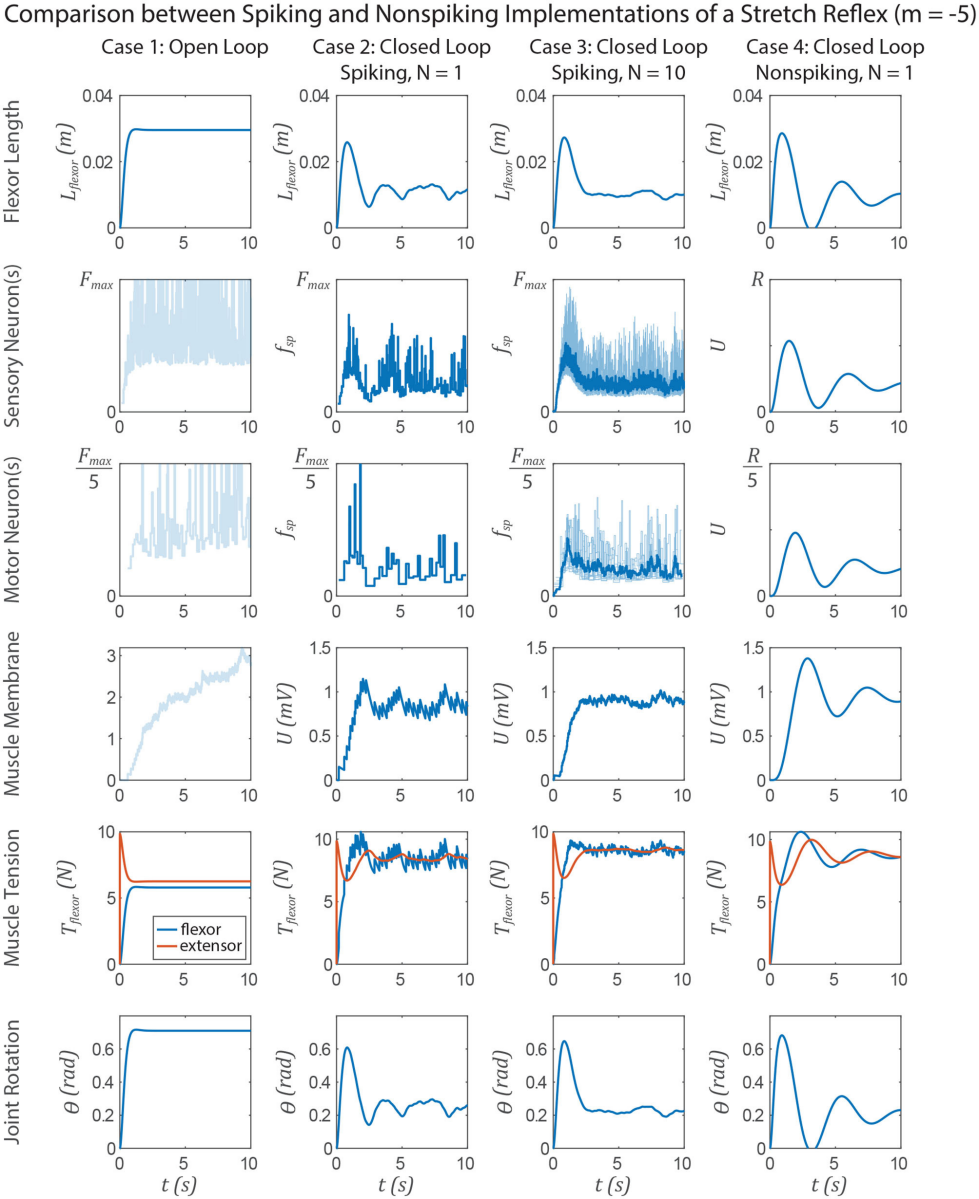
Data from a simulation of the system in Figure 1. Each column shows data from a different configuration of the system, from left to right: Case 1, open loop; case 2, spiking reflex pathway, *N* = 1; case 3, spiking reflex pathway, *N* = 10 (*f*_*sp*_ for every neuron is shown in light blue, population mean *f*_*sp*_ is plotted in dark blue); case 4, non-spiking reflex pathway. All corresponding axes are scaled the same. Each row plots data from a different stage of the reflex loop as depicted in Figure 1. In this figure, all spiking neurons have a variable spiking threshold θ (i.e., *m* = −5).

The similarities between non-spiking models and a population of spiking models apply to neuromechanical control models. Figures 11, 12 show data from a simulation of the muscle stretch reflex illustrated in Figure 1. In each case, the extensor muscle was activated, causing the flexor to stretch. The system's behavior was simulated in four scenarios: (1) open loop, that is, the flexor does not activate, although it can develop passive tension; (2) closed loop, with a pathway containing a single spiking neuron at each level that mediates a stretch reflex to activate the flexor and resist the stretch imposed by the extensor; (3) closed loop, with a pathway built from populations of 10 spiking neurons per node, connected as illustrated in Figure 8, and (4) closed loop, with a pathway built from non-spiking neurons and synapses. Noise was added to the model via reset noise (Gerstner, 2000). The impact of such reset noise on a neuron's encoding properties is calculated in Supplementary Materials (S1.1.6), and the model's formulation and parameter values are listed in Supplementary Materials (S1.2.1).

Figure 11 compares the system performance when the spiking neurons have constant spiking threshold θ, i.e., *m* = 0. In this case, there is effectively no transient spiking frequency when a stimulus is applied based on the length of the flexor muscle (Figure 1). In case 1, the open loop case, contracting the extensor results in significant joint extension, ≈0.7 radian. In all three closed loop cases, the joint extends much less in response to the muscle force due to the stretch reflex. After the major motions have died out, the muscle force, and therefore the joint angle, fluctuates the most in case 2, less in case 3, and not at all in case 4. All of the key response characteristics, including the angle overshoot, the settling time, and the final angle are the same in cases 2–4, suggesting that these systems are interchangeable models of such a stretch reflex.

Figure 12 compares the system performance when the spiking neurons do not have constant spiking threshold θ, i.e., *m* = −5. Tuning the spiking and non-spiking networks to exhibit similar transient behavior (e.g., in section 3.4) will result in much longer membrane time constants for the non-spiking system. In this case one would expect delayed activation of the nodes along the reflex pathway, causing a delayed muscle membrane depolarization and resulting in more overshoot and a longer rise time than when *m* = 0. Indeed this occurs in each case 2–4 relative to the experiments in Figure 11.

However, the non-spiking system in case 4 exhibits damped oscillation after case 3 has effectively come to rest. The spiking pathway does not exhibit such oscillations because the transient spiking frequency is due to the threshold θ changing from its *initial* value θ_0_ to the steady state value at which spikes occur $\theta_{\infty }^{*}$. Figure 2D illustrates this point, showing that once a neuron begins spiking, the value of θ^*^ is largely constant, even as the input *I*_*app*_ (and thus the spiking frequency *f*_*sp*_) increases. This is because the average voltage *U*_*avg*_ is relatively insensitive to changes in spiking frequency (Figure S1), causing only small changes in θ^*^ and thus *f*_*sp*_. Despite this mismatch in transient responses, the FSA enables us to rapidly construct models that contain both spiking and non-spiking elements.

## 5. Discussion

### 5.1. Summary

The analysis and numerical results in this manuscript show how continuous, non-spiking leaky-integrator neural dynamics can approximate the dynamics of a population of identical GLIF spiking neurons with randomized interconnections. The parallels in encoding and information transfer manifest through three analogs: (1) A spiking population's mean spiking frequency is analogous to the membrane voltage of a leaky integrator, (2) the transient spiking frequency of a spiking neuron can mimic a non-spiking neuron's transient voltage, and (3) a spiking synapse's average conductance is proportional to the pre-synaptic neuron's spiking frequency, analogous to how a non-spiking synapse's conductance is proportional to the pre-synaptic neuron's membrane depolarization. Since the dynamics are approximately similar, a network built from either type can be used to encode and transfer information in an equivalent manner. Therefore, networks of either type can have similar overall behavior, and either type might effectively be used to model and understand the nervous system.

The parallels between non-spiking neural models and models of populations of spiking neurons have been known for quite some time (Wilson and Cowan, 1972). However, the process and tools needed to set parameters within networks of these models to achieve desired and/or equivalent behavior have been lacking. In an attempt to apply the classical analysis from (Wilson and Cowan, 1972) to the practical application of programming neuromorphic hardware, we extended our functional subnetwork approach (FSA) to tuning networks of GLIF neurons and synapses. This extension enables one to tune control systems built from either non-spiking nodes or populations of spiking neurons. We presented a step-by-step method for tuning practical networks of populations of spiking neurons and synapses. We provided examples showing how increasing the number of neurons makes data transmission more ideal (i.e., match the expected population average activity). Finally, we provided a practical example of how such a method can be used to tune a neuromechanical model for control, and how the non-spiking and spiking implementations compare and contrast.

Despite the similarities between the models' activation in response to inputs (section 3.1) and how this activation maps to synaptic conductivity (section 3.3), we observe differences in the models' transient responses. A spiking neuron's smoothed (i.e., time-averaged) transient spiking frequency is a good match for a non-spiking neuron's transient membrane voltage if the spiking neuron is not initially spiking (Figures 6, 10). This is because the spiking threshold must change from the initial value θ_0_ to the steady state value when a spike occurs, $\theta_{\infty }^{*}$. However, the spike-time threshold $\theta_{\infty }^{*}$ is insensitive to a neuron's input current (and therefore the neuron's spiking frequency), meaning that the amplitude of the transient is very small once a neuron is spiking. This is not true for the non-spiking model, whose transient amplitude depends strongly on the input current. Therefore, the response properties of networks tuned to have long, exaggerated transient responses are a good match initially, but not once a neuron is already spiking (Figure 12). In all other respects, however, these models can be tuned to produce the same responses.

### 5.2. Expanding These Methods

The analysis in this manuscript primarily provides a framework for designing rate-coding networks, based on their steady-state spiking frequency. We have already shown how steady-state analysis contributes to designing arithmetic and dynamic (i.e., differentiation and integration over time) networks (Szczecinski et al., 2017b). However, not all neural computation is rate-coding, meaning that additional techniques are needed to expand this work to engineer direct encoding of other signals to produce more sophisticated neural behaviors. For instance, a non-spiking model captures class II excitability, in which a neuron's spiking frequency exponentially approaches a steady-state spiking frequency when subjected to a step input (Mihalaş and Niebur, 2009). However, the GLIF spiking neuron model used in this work can also exhibit class I excitability in which there is no transient spiking frequency, a behavior that a non-spiking neuron cannot replicate. Such a response might be useful in a reflex pathway, in which delayed sensory feedback may destabilize the system. In addition, a spiking neuron can exhibit phasic excitability, in which its spiking frequency starts high, but decays to 0 during a step input (Mihalaş and Niebur, 2009, see also Figure 3C). In the future, we plan to investigate whether such phasic responses could be used to replace the differentiation network from the non-spiking FSA approach (Szczecinski et al., 2017b) with a single neuron, a technique we have used in the past, but did not characterize thoroughly (Szczecinski et al., 2014). Exploiting single-neuron properties in this way could enable designers to pack more computational capability into chips with limited (albeit large) network sizes, such as Loihi (Davies et al., 2018).

However, creating small networks that seek to exploit single-neuron properties may reduce the accuracy of encoding, decoding, and other operations within the network. Alternative systems, such as the Neuroengineering Framework (NEF) (Eliasmith and Anderson, 2002), rely on nodes (ensembles) with many neurons (on the order of 10–1,000) to accurately encode information into the network. What makes NEF so powerful is the relatively hands-off design process, wherein the user specifies the intended function and number of neurons per node, and then NEF learns the intra-node parameter values necessary to perform that function (Eliasmith et al., 2012; Bekolay et al., 2014). This approach is much less onerous to the designer than the FSA, which requires explicit tuning of parameters for encoding, decoding, and other functions. We anticipate that these two approaches may complement one another, wherein the direct network tuning accomplished by the FSA could be used to initialize tuning within the NEF. In our experience, the FSA can be used to select initial network parameters that aid subsequent parameter optimization (Pickard et al., 2020). As a next step, we plan to compare the accuracy and efficiency of networks tuned via the FSA with those tuned via the NEF. We expect that the NEF may achieve arbitrarily high accuracy, but possibly at a computational cost. The FSA could be used to initialize learning networks in a less random way, requiring fewer neurons per node and less time to train.

### 5.3. When to Use Spiking or Non-spiking Neurons

A question that follows from this work is that if non-spiking and spiking neuron dynamics have many parallels, how does a modeler choose to use one or the other type? We believe that both types are useful in different contexts, depending on the knowledge available about the system to be modeled, the research question addressed by the model, and the real-world application of the network (e.g., in robotics).

A natural choice is to model spiking neurons in the nervous system with spiking models, and non-spiking neurons with non-spiking models. However, biomechanical constraints determine whether animals use spiking or non-spiking neurons. Specifically, action potentials can be transmitted over long distances, whereas graded (i.e., non-spiking) signals tend to dissipate over even short distances. This may be why many non-spiking neurons have been identified in insects and other small animals, particularly for integrating sensory information (Burrows et al., 1988; Sauer et al., 1996); they have less room in their bodies to house networks of many spiking neurons, and their small bodies do not require them to transmit information over long distances. No matter why animals have spiking or non-spiking neurons, a computer model does not share these biochemical constraints, so it is worth deciding how to model networks based on the computational hardware available.

One purely technological motivation to use spiking models is for model implementation on neuromorphic hardware. Chips, such as Loihi (Davies et al., 2018), SpiNNaker (Khan et al., 2008), TrueNorth (Merolla et al., 2014), and others (Pfeil et al., 2013; Gehlhaar, 2014; Ionica and Gregg, 2015) use non-traditional architecture to simulate hundreds of thousands of spiking neurons and hundreds of millions of synapses in real-time while using on the order of one watt of power. Neuromorphic computers tend to use spiking models because they have less communication overhead than non-spiking networks. For spiking networks, communication can be binary (1 bit per spike) and only must occur after a spike occurs, at a maximum of 200–300 Hz (Gerstner et al., 1997; Carter and Bean, 2010) but more often below 1–2 Hz (Kerr et al., 2005). In contrast, non-spiking synapses need to be updated during every simulation step, because they depend on the pre-synaptic neuron's continuous membrane voltage. Such a requirement significantly increases overhead relative to spiking models.

Spiking neurons and synapses are also attractive because they enable the use of spike timing dependent plasticity (STDP) learning techniques (Gerstner et al., 1996; Markram et al., 1997, 2012), a powerful class of machine learning tools. These methods have been applied to many stimulus-recognition tasks, such as hearing, speech, and vision. They measure the coincidence of incoming spikes to increase or decrease the strength of synapses in the network, and thus rely on spiking models. These networks are typically classified as “self-organizing,” meaning that they initially have no structure, but develop their own structure as connections are pruned due to disuse. However, many parts of the nervous system have exquisite structure, and lend themselves to being directly modeled, structure, and all. Thus, we believe that the methods in this manuscript may serve to produce baseline parameter values for a highly structured network, which may then use spike-based mechanisms to tune itself over time, a technique like that utilized in Nengo (Bekolay et al., 2014). In such cases, populations of spiking neurons would be preferable to non-spiking population models, but could be initialized using the tuning rules presented in this manuscript.

Additionally, spiking neurons and synapses may be beneficial because even a simple model like that used in this work can produce a wide variety of behaviors and responses (Mihalaş and Niebur, 2009). For example, setting *m* > 0 can produce spike frequency adaptation and phasic spiking, which are known to be critical for filtering sensory feedback in locomotory systems (Mamiya et al., 2018; Zill et al., 2018). Such rate-sensitive responses can be produced by small networks of non-spiking neurons and synapses (Szczecinski et al., 2017b), but force the modeler to use more neurons than may be necessary. Therefore, if the modeler knows that single neurons in their model system generate more complex responses than class I or II excitability, then spiking models should be utilized. However, if the neuron responses in the network are simple, then the model could be implemented as a network of non-spiking neurons instead.

We believe non-spiking networks may be particularly beneficial if a model is not meant to run on specialized neuromorphic hardware. Simulating the membrane voltage of each spiking neuron in a population requires storing and updating orders of magnitude more states than simply using a non-spiking node to represent the mean activity of the population. In addition, throughout this study, we observed that the timing of spikes was sensitive to the simulation step used (i.e., spikes cannot happen between time steps), and simulations only closely matched our analytic predictions as the time step became very small. We also tested adaptive stepping algorithms (Matlab's ode45 and ode15s solvers); however, the discontinuous nature of spikes required the use of event functions that halt simulation whenever a spike occurs, complicating the code. In general, we found that it took longer to simulate the dynamics of a spiking network relative to those of a non-spiking network. These reasons motivate implementing networks on traditional computers with non-spiking models.

Finally, non-spiking neurons may contribute to models by representing neuromodulatory neurons that cause large-scale changes to network behavior. In Szczecinski et al. (2017b), we not only designed “signal transmission” pathways as in this work, but also “signal modulation” pathways, in which one neuron could modify the gain of another neuron's response to its synaptic inputs. When we tested signal modulation pathways built from spiking neurons and synapses, the results were poor (data not shown). Due to the discrete nature of spikes, modulation was inconsistent, leading to unpredictably varying firing frequencies from the “modulated” neuron. However, it may be advantageous to instead construct networks in which signals are transmitted via spiking neurons, but modulated via non-spiking neurons. These non-spiking neurons in effect model neuromodulatory neurons, whose voltage reflects the concentration of a neuromodulator in the network, and whose non-spiking synapses represent the activation of receptors sensitive to that particular neuromodulator. Such pathways may change the resting potential, membrane conductance, and time constant of other neurons throughout the network (for a review, see Miles and Sillar, 2011). Such non-spiking neurons would have long time constants, enabling them to modify network performance on much longer timescales than that of a single spike. Such neurons could receive either descending input from the brain, or ascending input from local sensory neurons. The result would be a model that can modulate its control system based on exteroception and interoception, and exhibit truly adaptive, context-dependent behavior.

## Data Availability Statement

The raw data supporting the conclusions of this article will be made available by the authors, without undue reservation.

## Author Contributions

NS conceived of the study. NS, RQ, and AH planned the results to be collected. NS collected results and wrote the manuscript. RQ and AH edited the manuscript. All authors contributed to the article and approved the submitted version.

## Conflict of Interest

The authors declare that the research was conducted in the absence of any commercial or financial relationships that could be construed as a potential conflict of interest.
