# Supplementary material for: Extending the Functional Subnetwork Approach to a Generalized Linear Integrate-and-Fire Neuron Model
Source: Front Neurorobot. 2020 Nov 13;14:577804. doi: 10.3389/fnbot.2020.577804 (PMC7691602; doi:10.3389/fnbot.2020.577804)
Supplement: Supplementary file 1 [file Data_Sheet_1.pdf]

frontiers

Supplementary Material

1 SUPPLEMENTARY DATA

1.1 Relationships between U_∞ and f_{sp}

1.1.1 Complete solution for $\theta(t)$

Calculating f_{sp} requires that one first solve for the threshold during steady-state firing, $\theta(t)$, which is periodic at the spiking frequency. This condition is expressed as:

$$\theta(0) = \theta(0 + T_{sp}) = \theta^*_\infty, \tag{S1}$$

where θ^*_∞ is the constant value of θ at the instant a spike occurs in steady-state and $T_{sp} = 1/f_{sp}$. To calculate θ^*_∞, $\theta(t)$ is solved analytically, under the assumption that only one value of θ^*_∞ will satisfy Eq. S1, given the neuron's properties and inputs.

θ has linear dynamics, so its response has a complementary and particular component, where

$$\theta(t) - \theta_\infty = \theta_{comp} + \theta_{part}. \tag{S2}$$

θ_∞ is computed from Eq. 12. The complementary solution θ_{comp} is simply

$$\theta_{comp} = A \cdot e^{-t/\tau_\theta}, \tag{S3}$$

where A is an undetermined coefficient calculated based on the initial conditions of the total response. The particular response $\theta_{part}(t)$ is found by convolving $f(t)$ (the transient part of $U(t)$) with θ's impulse response, $g(t)$. These terms are:

$$f(t) = -m \cdot U_\infty \cdot e^{-t/\tau_{mem}} \tag{S4}$$

$$g(t) = \frac{1}{\tau_\theta} \cdot e^{-t/\tau_\theta}. \tag{S5}$$

The definition of the convolution can be applied to compute

$$\theta_{part}(t) = f(t) * g(t) = \int_0^t -m \cdot U_\infty \cdot e^{-s/\tau_{mem}} \cdot \frac{1}{\tau_\theta} \cdot e^{-(t-s)/\tau_\theta} \cdot ds. \tag{S6}$$

Removing constant terms from the integrand,

$$\theta_{part}(t) = \frac{-m \cdot U_\infty}{\tau_\theta} \cdot e^{-t/\tau_\theta} \cdot \int_0^t e^{-s/\tau_{mem}} \cdot e^{s/\tau_\theta} \cdot ds. \tag{S7}$$

If $\tau_{mem} \neq \tau_\theta$, then the exponentials in the integrand are combined, integrated, and reseparated such that

$$\theta_{part}(t) = m \cdot U_\infty \cdot \frac{\tau_{mem}}{\tau_\theta - \tau_{mem}} \cdot \left(e^{-t/\tau_{mem}} - e^{-t/\tau_\theta}\right). \tag{S8}$$

If $\tau_{mem} = \tau_\theta$, then Eq. S8 gives an undefined $0/0$ result. This is rectified by consulting Eq. S7 and recognizing that the integrand equals 1 in this case. Thus, the particular solution is

$$\theta_{part}(t) = \begin{cases} -\frac{m \cdot U_\infty}{\tau_\theta} \cdot t \cdot e^{-t/\tau_\theta}, & \text{if } \tau_{mem} = \tau_\theta, \\ m \cdot U_\infty \cdot \frac{\tau_{mem}}{\tau_\theta - \tau_{mem}} \cdot \left(e^{-t/\tau_{mem}} - e^{-t/\tau_\theta}\right), & \text{else.} \end{cases} \tag{S9}$$

Now that $\theta_{part}(t)$ is known, the initial condition is used to solve for A in Eq. S3. Recall that the initial condition is the unknown value θ^*_∞. Also, note that no matter the values of τ_{mem} and τ_θ, $\theta_{part}(t = 0) = 0$. Evaluating Eq. S2 when $t = 0$ and substituting in Eq. 12,

$$A = \theta^*_\infty - (\theta_0 + m \cdot U_\infty). \tag{S10}$$

The total response of θ is found using Eq. S2. θ_∞ is defined in Eq. 12. $\theta_{comp}(t)$ is defined in Eq. S3, where the value of A is calculated in Eq. S10. $\theta_{part}(t)$ is the piecewise function in Eq. S9. Combining all of these yields the full solution for the spiking threshold over time,

$$\theta(t) = \theta_0 + m \cdot U_\infty + (\theta^*_\infty - \theta_0 - m \cdot U_\infty) \cdot e^{-t/\tau_\theta} + \begin{cases} -\frac{m \cdot U_\infty}{\tau_\theta} \cdot t \cdot e^{-t/\tau_\theta}, & \text{if } \tau_{mem} = \tau_\theta, \\ m \cdot U_\infty \cdot \frac{\tau_{mem}}{\tau_\theta - \tau_{mem}} \cdot \left(e^{-t/\tau_{mem}} - e^{-t/\tau_\theta}\right), & \text{else.} \end{cases} \tag{S11}$$

1.1.2 Solving for θ^*_∞

To solve for θ^*_∞, the condition $\theta(T_{sp}) = \theta^*_\infty$ is applied to Eq. S11 and solved. $T_{sp} = 1/f_{sp}$ could be simply substituted into Eq. S11; however, t usually appears in Eq. S11 in the form e^t. Therefore, let us instead solve Eq. 13 for the following terms, to be substituted into Eq. S11:

$$e^{-T_{sp}/\tau_{mem}} = 1 - \frac{\theta^*_\infty}{U_\infty} \tag{S12}$$

$$e^{-T_{sp}/\tau_\theta} = \left(1 - \frac{\theta^*_\infty}{U_\infty}\right)^{\tau_{mem}/\tau_\theta}. \tag{S13}$$

Setting $t = T_{sp}$ and substituting Eqs. 12, S12, and S13 into Eq. S11,

$$\theta(T_{sp}) = \theta^*_\infty = \theta_\infty + (\theta^*_\infty - \theta_\infty) \cdot \left(1 - \frac{\theta^*_\infty}{U_\infty}\right)^{\tau_{mem}/\tau_\theta} + \begin{cases} m \cdot U_\infty \cdot \frac{\tau_{mem}}{\tau_\theta} \cdot \ln\left(1 - \frac{\theta^*_\infty}{U_\infty}\right) \cdot \left(1 - \frac{\theta^*_\infty}{U_\infty}\right)^{\tau_{mem}/\tau_\theta}, & \text{if } \tau_{mem} = \tau_\theta, \\ m \cdot U_\infty \cdot \frac{\tau_{mem}}{\tau_\theta - \tau_{mem}} \cdot \left(1 - \frac{\theta^*_\infty}{U_\infty} - \left(1 - \frac{\theta^*_\infty}{U_\infty}\right)^{\tau_{mem}/\tau_\theta}\right), & \text{else.} \end{cases} \tag{S14}$$

Equation S14 can be simplified by grouping like terms and simplifying $\tau_{mem}/\tau_\theta = 1$ in the top line. However, the result will be an implicit, transcendental function, which can only be solved numerically. One method is to set Eq. S14 equal to 0 and use a root-finding algorithm to find θ^*_∞ that satisfies the resulting

function,

$$f(\theta^*_\infty) = 0 = \begin{cases} (\theta_\infty - \theta^*_\infty) \cdot \frac{\theta^*_\infty}{U_\infty} + m \cdot U_\infty \cdot \left(1 - \frac{\theta^*_\infty}{U_\infty}\right) \cdot \ln\left(1 - \frac{\theta^*_\infty}{U_\infty}\right), & \text{if } \tau_{mem} = \tau_\theta, \\ (\theta_\infty - \theta^*_\infty) \cdot \left(1 - (1 - \frac{\theta^*_\infty}{U_\infty})^{\tau_{mem}/\tau_\theta}\right) + \\ \frac{m \cdot U_\infty \cdot \tau_{mem}}{\tau_\theta - \tau_{mem}} \cdot \left((1 - \frac{\theta^*_\infty}{U_\infty}) - (1 - \frac{\theta^*_\infty}{U_\infty})^{\tau_{mem}/\tau_\theta}\right), & \text{else.} \end{cases} \tag{S15}$$

1.1.3 $U_{avg} \to \theta^*/2$ as f_{sp} increases

As U_∞ increases, $U_{avg} \to \theta^*/2$. This is because the neuron spikes when $U = \theta^*_\infty$, and then its voltage is reset to 0 (Fig. S1). In addition, the neuron voltage integrates more quickly when U_∞ is large. Thus, the neuron voltage over time approaches a sawtooth waveform with a maximum value of θ^*_∞ as U_∞ increases. The mean value of such a waveform is $\theta^*_\infty/2$.

1.1.4 f_{sp} can be bounded by parallel lines

The spiking frequency of the GLIF neuron can be approximated by an affine function of I_{app}, and can be bounded by affine functions. A property of the natural logarithm is

$$\frac{x-1}{x} \leq \ln x \leq x - 1. \tag{S16}$$

The center term is set equal to the spiking frequency f_{sp} (Eq. 13) by substituting $x = 1 - \theta^*_\infty/U_\infty$, then inverting the equation:

$$\frac{U_\infty - \theta^*_\infty}{\tau_{mem} \cdot \theta^*_\infty} \leq \frac{-1}{\tau_{mem} \cdot \ln\left(1 - \frac{\theta^*_\infty}{U_\infty}\right)} \leq \frac{U_\infty}{\tau_{mem} \cdot \theta^*_\infty} \tag{S17}$$

Note that the sense of the inequality does not change because the equation is made negative, and then inverted.

The expression in the center is now equal to f_{sp}. Equation S17 shows that the spiking frequency can be bounded by parallel lines,

$$\frac{U_\infty - \theta^*_\infty}{\tau_{mem} \cdot \theta^*_\infty} \leq f_{sp}(U_\infty, \theta^*_\infty) \leq \frac{U_\infty}{\tau_{mem} \cdot \theta^*_\infty}. \tag{S18}$$

Substituting Eq. 3 for U_∞ and setting $\bar{G}_s = 0$, the spiking frequency is bounded by two affine functions of the applied current I_{app},

$$\frac{\frac{I_{app}+I_{bias}}{G_{mem}} - \theta^*_\infty}{\tau_{mem} \cdot \theta^*_\infty} \leq f_{sp}(I_{app}, \theta^*_\infty) \leq \frac{\frac{I_{app}+I_{bias}}{G_{mem}}}{\tau_{mem} \cdot \theta^*_\infty}. \tag{S19}$$

If

$$I_{bias} = \frac{G_{mem} \cdot \theta^*_\infty}{2}, \tag{S20}$$

then

$$\frac{I_{app}}{G_{mem} \cdot \tau_{mem} \cdot \theta^*_\infty} - \frac{1}{2 \cdot \tau_{mem}} \leq f_{sp}(I_{app}, \theta^*_\infty) \leq \frac{I_{app}}{G_{mem} \cdot \tau_{mem} \cdot \theta^*_\infty} + \frac{1}{2 \cdot \tau_{mem}}. \tag{S21}$$

For any U_∞, the precise value of θ^*_∞ can be calculated numerically with Eq. S11, or approximated with the explicit function in Eq. 16. Then, Eq. S18 provides affine bounds on the spiking frequency, with a range of

$1/\tau_{mem}$. f_{sp} approaches the mean of the bounds in Eq. S18, providing the approximation for f_{sp},

$$f_{sp,approx}(I_{app}, \theta^*_\infty) = \frac{I_{app}}{G_{mem} \cdot \tau_{mem} \cdot \theta^*_\infty}. \tag{S22}$$

1.1.5 Solving for the evolution of the spiking threshold, $\theta^*(t)$

Setting $U = \theta/2$ in Eq. 9 and rearranging,

$$\tau_\theta \cdot \frac{d\theta^*}{dt} + \left(1 - \frac{m}{2}\right) \cdot \theta^* = \theta_0. \tag{S23}$$

Substituting in B from Eq. 16,

$$\tau_\theta \cdot B \cdot \frac{d\theta^*}{dt} + \theta^* = B \cdot \theta_0. \tag{S24}$$

The steady-state solution to Eq. S24, θ^*_∞, is the solution when $d\theta^*/dt = 0$,

$$\theta^*_\infty = B \cdot \theta_0, \tag{S25}$$

which is consistent with Eq. 16. Given the form of Eq. S24, the complementary response is

$$\theta^*_{comp}(t) = A \cdot e^{-t/\tau_{\theta^*}}, \tag{S26}$$

where $\tau_{\theta^*} = \tau_\theta \cdot B$. Applying the initial condition $\theta^*(0) = \theta_0$, the full solution is

$$\theta^*(t) = B \cdot \theta_0 + \theta_0 \cdot (1 - B) \cdot e^{-t/\tau_{\theta^*}}. \tag{S27}$$

Substituting Eq. S25 into Eq. S27,

$$\theta^*(t) = \theta^*_\infty + (\theta_0 - \theta^*_\infty) \cdot e^{-t/\tau_{\theta^*}}. \tag{S28}$$

1.1.6 Impact of reset noise on f_{sp}

The impact of reset noise on f_{sp} can be determined by modifying the analysis in the previous section. Reset noise is one way to add stochasticity to a neuron's firing frequency. In this paradigm, the neuron voltage U is not reset to 0 after it spikes. Instead, it is reset to X, a random variable selected from probability distribution $f_X(x)$, which has a mean of 0 and $||f_X(x)|| \to 0$ as $||x|| \to \infty$. For simplicity, we assume that $||X|| \ll \theta$.

Given the stochastic reset voltage X, the membrane voltage evolution after a spike from Eq. 5 becomes

$$U(t) = U_\infty \cdot (1 - e^{-t/\tau_{mem}}) + X \cdot e^{-t/\tau_{mem}} \tag{S29}$$

It is immediately apparent from Eq. S29 that the effect of the reset noise decays over time from the reset event, implying that reset noise will alter the spiking frequency predominantly when interspike intervals are short, i.e. when $f_{sp} \gg 0$.

The effect of random reset on the spiking frequency is calculated by using Eq. S29 to compute the spiking frequency,

$$f_{sp} = Y = \frac{-1}{\tau_{mem} \cdot \ln\left(1 - \frac{\theta - X}{U_\infty - X}\right)}, \tag{S30}$$

where X is the randomly selected reset voltage after the previous spike and Y is the subsequent spiking frequency, which is now a random variable. If we label this function in Eq. S30 $Y = g(X)$, then the distribution of spiking frequencies given the reset noise is

$$f_Y(Y = y) = f_X\left(g^{-1}(y)\right) \cdot \left\|\frac{\partial}{\partial y} g^{-1}(y)\right\|. \tag{S31}$$

Since $g(\cdot)$ monotonically increases when $ln(\cdot)$ is defined, $g^{-1}(\cdot)$ is defined over the same interval,

$$g^{-1}(y) = U_\infty - (U_\infty - \theta) \cdot e^{1/\tau_{mem} \cdot y}. \tag{S32}$$

Analytically carrying out the calculation in Eq. S31 is cumbersome. To demonstrate that the distribution of possible spiking frequencies f_{sp} increases as the applied current increases, Fig. S2 plots the result of numerically computing Eq. S31 given the distribution of reset voltage used in Sec. 4 (Fig. S2A). One can see that for particular values of applied current I_{app}, the distribution of spiking frequencies widens as the spiking frequency increases (Fig. S2B). Computing the range of spiking frequencies across the entire continuum of applied current values further demonstrates this trend (Fig. S2C).

For a more detailed treatment of the effect of reset noise on an integrate-and-fire neuron's spike timing, see Sec. 2.2.2 of (Gerstner, 2000).

1.2 Parameter values for neuromechanical simulation

1.2.1 Mechanical and muscular parameter values

The model and parameter values were taken from (Yu and Wilson, 2012). A human forearm, bicep (flexor), and tricep (extensor) were simulated. Each muscle was modeled as a linear Hill muscle with no length-tension relationship. The resulting system has 4 dynamical variables: The joint angle θ, the joint angular velocity $\dot{\theta}$, the flexor tension T_{flx}, and the extensor tension T_{ext}. The system also has two inputs, F_{ext} and F_{flx}, which are activation of the extensor and flexor muscles, respectively. The dynamics are described by the following state equations:

$$J \cdot \ddot{\theta} = M_{ext} - M_{flx} - c_{joint} \cdot \dot{\theta} \tag{S33}$$

$$\frac{dT_{ext}}{dt} = \frac{k_{se,ext}}{c_{ext}} \cdot \left(k_{pe,ext} \cdot (\|\vec{L}_{ext}\| - L_{rest,ext}) + c_{ext} \cdot \frac{d}{dt}\|\vec{L}_{ext}\| - \left(1 + \frac{k_{pe,ext}}{k_{se,ext}}\right) \cdot T_{ext} + F_{ext} \right) \tag{S34}$$

$$\frac{dT_{flx}}{dt} = \frac{k_{se,flx}}{c_{flx}} \cdot \left(k_{pe,flx} \cdot (\|\vec{L}_{flx}\| - L_{rest,flx}) + c_{flx} \cdot \frac{d}{dt}\|\vec{L}_{flx}\| - \left(1 + \frac{k_{pe,flx}}{k_{se,flx}}\right) \cdot T_{flx} + F_{flx} \right). \tag{S35}$$

The joint angle and muscle tension are related by the geometry of muscle attachments. When the joint rotates, the muscle lengths change; when the muscle lengths change, the muscle moments M_{ext} and M_{flx} change; and when the muscle moments change, the joint rotates. Specifically, the positions of the muscle attachments on the distal limb segment relative to the joint are calculated by:

$$\vec{r}_{ext} = \begin{bmatrix} \cos(\theta) & \sin(\theta) \\ -\sin(\theta) & \cos(\theta) \end{bmatrix} \cdot \begin{bmatrix} -r \\ 0 \end{bmatrix}$$

$$\vec{r}_{flx} = \begin{bmatrix} \cos(\theta) & \sin(\theta) \\ -\sin(\theta) & \cos(\theta) \end{bmatrix} \cdot \begin{bmatrix} r \\ 0 \end{bmatrix}.$$

The lines of action of the muscles are calculated by:

$$\vec{L}_{ext} = \begin{bmatrix} -r \\ L \end{bmatrix} + \vec{r}_{ext}$$

$$\vec{L}_{flx} = \begin{bmatrix} r \\ L \end{bmatrix} + \vec{r}_{flx}$$

The moment that each muscle applies to the limb is the cross product between that muscle's moment arm r and the muscle's line of action, multiplied by the tension at that instant:

$$M_{ext} = \left\| \vec{r}_{ext} \times \frac{\vec{L}_{ext}}{\|\vec{L}_{ext}\|} \right\| \cdot T_{ext} \tag{S36}$$

$$M_{flx} = \left\| \vec{r}_{flx} \times \frac{\vec{L}_{flx}}{\|\vec{L}_{flx}\|} \right\| \cdot T_{flx}. \tag{S37}$$

When the inputs $F_{ext} = 0$ and $F_{flx} = 0$, the system has a stable equilibrium point when all states equal 0. Altering F_{ext} and/or F_{flx} will change the equilibrium muscle tension and joint angle, but the equilibrium point remains stable. This is because the muscles act like springs, passively applying a reaction torque to correct small changes in joint angle. The parameter values of the model are listed in Tab. S1.

For both of the pathways in Sec. 4, $R = 20$ mV, $F_{max} = 100$ Hz,

$$I_{app} = L_{flexor} \cdot \frac{R}{r}, \tag{S38}$$

and

$$F_{flexor} = 15\text{ N} \cdot \begin{cases} 0, & \text{if } \bar{U}_{muscle} \leq 0, \\ \frac{\bar{U}_{muscle}}{R}, & \text{if } 0 < \bar{U}_{muscle} < R, \\ 1, & \text{if } \bar{U}_{muscle} >\geq R. \end{cases} \tag{S39}$$

The rest of the parameter values are listed in Tabs. S2 and S3.

2 SUPPLEMENTARY TABLES AND FIGURES

2.1 Tables

Table S1. Table of muscular and mechanical parameter values

Parameter	Description	Value
L [m]	forearm length	250×10^{-3}
c_{joint} [Nm(ms)/rad]	joint damping	200
J [Nm(ms)^2/rad]	forearm mass moment of inertia	27.9×10^3
r [m]	muscle moment arm	45×10^{-3}
$k_{pe,flx}$ [N/m]	flx. parallel elastic element stiffness	200
$k_{se,flx}$ [N/m]	flx. series elastic element stiffness	10×10^3
c_{flx} [Nm(ms)/rad]	flx. parallel element damping	39.5
$L_{rest,flx}$ [m]	flx. resting length	250×10^{-3}
$k_{pe,ext}$ [N/m]	ext. parallel elastic element stiffness	126
$k_{se,ext}$ [N/m]	ext. series elastic element stiffness	10×10^3
c_{ext} [Nm(ms)/rad]	ext. parallel element damping	1990
$L_{rest,ext}$ [m]	ext. resting length	250×10^{-3}

Table S2. Table of neural and synaptic parameter values (accompanies Fig. 11)

**Parameter**	**Case 2**	**Case 3**	**Case 4**
Sensory Neuron			
N	1	10	1
τ_{mem} or $\bar{\tau}_{mem}$ [ms]	200	200	500
m	0	0	N/A
θ_0 [mV]	1	1	N/A
τ_θ [ms]	N/A	N/A	N/A
I_{bias} [nA]	0.5	0.5	0
Synaptic Connection			
k_{syn}	0.2	0.2	0.2
G_{max} or $\bar{G}_{max}$ [μS]	118×10^{-3}	11.8×10^{-3}	25.6×10^{-3}
E_s [mV]	160	160	160
τ_s [ms]	2.17	2.17	N/A
Motor Neuron(s)			
N	1	10	1
τ_{mem} or $\bar{\tau}_{mem}$ [ms]	200	200	500
m	0	0	N/A
θ_0 [mV]	1	1	N/A
τ_θ [ms]	N/A	N/A	N/A
I_{bias} [nA]	0.5	0.5	0
Muscular Junction			
k_{syn}	1	1	1
G_{max} or $\bar{G}_{max}$ [μS]	658×10^{-3}	65.8×10^{-3}	143×10^{-3}
E_s [mV]	160	160	160
τ_s [ms]	2.17	2.17	N/A
Muscle Membrane			
N	1	1	1
τ_{mem} or $\bar{\tau}_{mem}$ [ms]	1500	1500	1500
m	N/A	N/A	N/A
θ_0 [mV]	N/A	N/A	N/A
τ_θ [ms]	N/A	N/A	N/A
I_{bias} [nA]	0	0	0

Table S3. Table of neural and synaptic parameter values (accompanies Fig. 12)

**Parameter**	**Case 2**	**Case 3**	**Case 4**
Sensory Neuron			
N	1	10	1
τ_{mem} or $\bar{\tau}_{mem}$ [ms]	700	700	500
m	-5	-5	N/A
θ_0 [mV]	1	1	N/A
τ_θ [ms]	1750	1750	N/A
I_{bias} [nA]	0.143	0.143	0
Synaptic Connection			
k_{syn}	0.2	0.2	0.2
G_{max} or $\bar{G}_{max}$ [μS]	118×10^{-3}	11.8×10^{-3}	25.6×10^{-3}
E_s [mV]	160	160	160
τ_s [ms]	2.17	2.17	N/A
Motor Neuron(s)			
N	1	10	1
τ_{mem} or $\bar{\tau}_{mem}$ [ms]	700	700	500
m	-5	-5	N/A
θ_0 [mV]	1	1	N/A
τ_θ [ms]	1750	1750	N/A
I_{bias} [nA]	0.143	0.143	0
Muscular Junction			
k_{syn}	1	1	1
G_{max} or $\bar{G}_{max}$ [μS]	658×10^{-3}	65.8×10^{-3}	143×10^{-3}
E_s [mV]	160	160	160
τ_s [ms]	2.17	2.17	N/A
Muscle Membrane			
N	1	1	1
τ_{mem} or $\bar{\tau}_{mem}$ [ms]	1500	1500	1500
m	N/A	N/A	N/A
θ_0 [mV]	N/A	N/A	N/A
τ_θ [ms]	N/A	N/A	N/A
I_{bias} [nA]	0	0	0

2.2 Figures

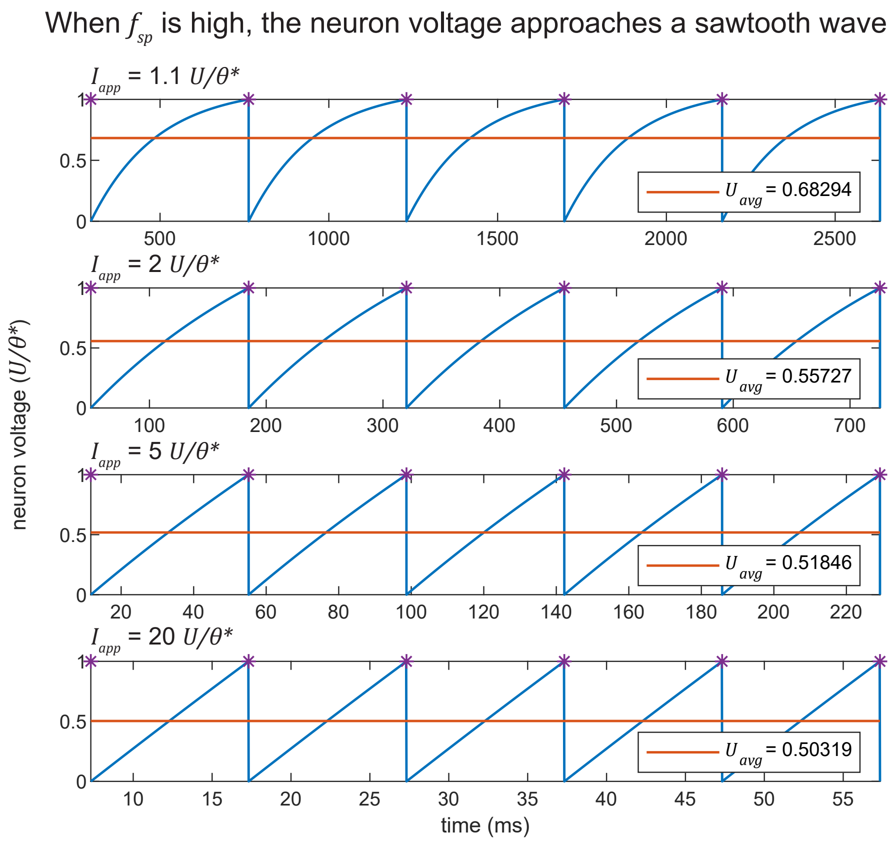

Figure S1. At high firing frequencies, the neuron voltage time course resembles a sawtooth wave. Each plot shows $U(t)$ (blue) given a larger I_{app}, and thus a higher f_{sp}. Spikes are indicated by violet stars. The average voltage U_{avg} is plotted in red.

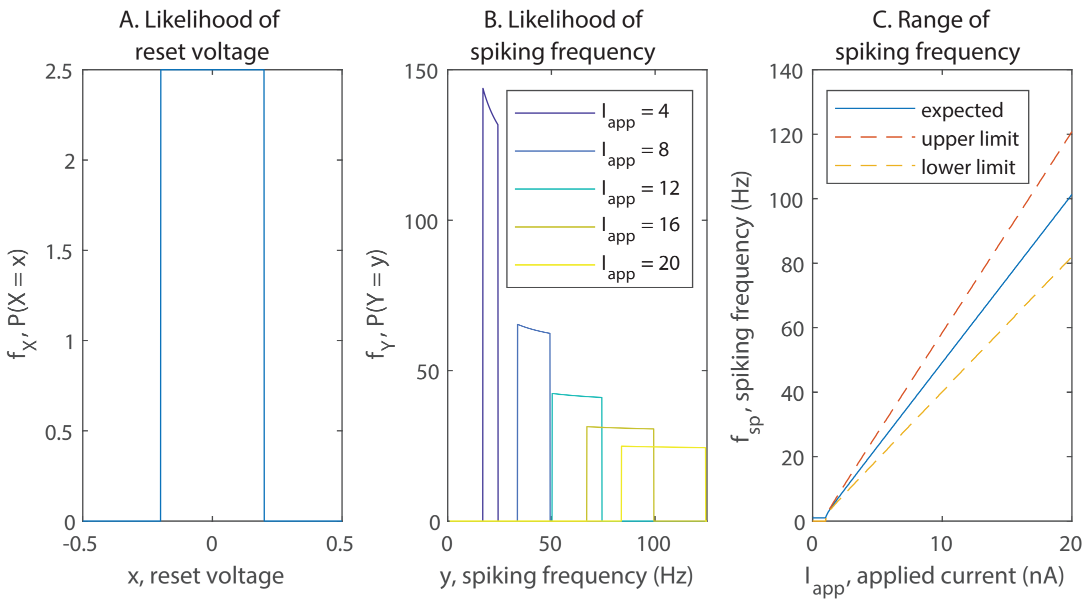

Figure S2. As the spiking frequency increases, the impact of reset noise increases. Given the uniform random reset voltage after spikes A) and following Eq. S31, the likelihood of spiking frequency can be calculated B). As the applied current increases, the distribution of possible spiking frequencies increases C), as predicted in the Supplementary Document (S1.1.6).

REFERENCES

W. Gerstner, Population Dynamics of Spiking Neurons: Fast Transients, Asynchronous States, and Locking, Neural Computation 12 (2000) 43–89.

T. F. Yu, A. J. Wilson, A novel passive movement method for parameter estimation of a musculoskeletal arm model incorporating a modified Hill muscle model, IFAC Proceedings Volumes (IFAC-PapersOnline) 45 (2012) 166–171.
